# DNA-based monitoring uncovers contrasting salinity-driven diversity patterns of bacterial and protist plankton in the Baltic Sea area

**DOI:** 10.1038/s42003-026-10977-4

**Published:** 2026-10-07

**Authors:** Krzysztof T. Jurdzinski, Meike AC Latz, Anders Torstensson, Sonia Brugel, Mikael Hedblom, Yue O. O. Hu, Markus Lindh, Agneta Andersson, Bengt Karlson, Anders F. Andersson

**Affiliations:** 1 KTH Royal Institute of Technology, Department of Gene Technology, Science for Life Laboratory, Stockholm, Sweden; 2 University of Copenhagen, Department of Plant and Environmental Sciences, Frederiksberg C, Denmark; 3 Swedish Meteorological and Hydrological Institute, Västra Frölunda, Sweden; 4 Umeå University, Department of Ecology, Environment and Geoscience, Umeå, Sweden; 5 Umeå Marine Sciences Centre, Umeå University, Norrbyn, Sweden; 6 Present Address: State Key Laboratory of Ecological Safety and Sustainable Development in Arid Lands, Xinjiang Institute of Ecology and Geography, Chinese Academy of Sciences, Urumqi, China; 7 Present Address: University of Chinese Academy of Sciences, Beijing, China

**Keywords:** Microbial ecology, Microbial biooceanography, Water microbiology, Biodiversity, Biogeography

## Abstract

Salinity has been identified as a primary factor structuring community composition in global diversity assessments of free-living bacteria in non-extreme environments. It remains unknown whether the same is true for protists. Moreover, changes in alpha diversity of both bacteria and protists across the brackish salinity gradient remain elusive. Here, we analyzed protist and bacterial diversity using DNA metabarcoding across 398 surface water samples synchronized with national monitoring of the Baltic Sea and the Kattegat-Skagerrak. We show that salinity, unlike other environmental factors, had a stronger effect on bacterial than on protist community composition. Likewise, protist taxonomic units tended to have broader salinity niches than bacterial ones. In contrast, protist but not bacterial alpha diversity (richness) increased with salinity. Bacterial alpha diversity was similar across salinities despite drastic changes in community composition, as marine-associated taxa steadily replaced freshwater-associated ones. Conversely, marine-associated protists dominated across most of the salinity gradient, with fewer taxonomic units adapted to lower salinities. Overall, the diversity of protists but not bacteria followed patterns consistent with local adaptation as observed in other groups of organisms. Ultimately, the observed patterns form a baseline for environmental monitoring and suggest unexplored differences between bacterial and protist eco-evolutionary dynamics.

## Introduction

Since the earliest global surveys of bacterial diversity, salinity has emerged as the strongest factor structuring the community composition of free-living bacteria^1–4^. It is well established that freshwater-marine transitions have been infrequent events in microbial evolution, separating bacterial as well as protist communities^5–7^. However, the freshwater-marine distinction is a binary one, omitting the brackish salinities in between (0.5-30 PSU). Brackish waters comprise a volume at least comparable with that of global lakes^8–10^, and are mostly concentrated in coastal zones and inland lakes, areas of markedly high importance for and pressure from human activities. Moreover, these ecosystems are highly dependent on microorganisms, which are responsible for most of the primary production and decomposition in these ecosystems^11^. Finally, brackish waters host a distinct microbiome, with a vast majority of bacterial species lacking representatives in freshwater and marine environments^8,12^.

A number of studies have assessed the impact of salinity on bacterial^13–22^ and protist^16,18,21–25^ plankton communities in brackish waters. Generally, a pronounced shift in community composition along the salinity gradient has been observed for both bacteria and protists. Bacterial community composition changes more with salinity than with season^15,17^, but it remains unclear whether the same is true for protists. A few studies have compared beta diversity patterns of bacterial and protist brackish plankton^16,18,21,22^, yet it is not established whether the community composition of both microbial groups is similarly affected by abiotic factors. Results obtained across steep salinity gradients in Antarctica suggested stronger environmental filtering among planktonic bacteria than protists^16,26^, while studies in other environmental contexts (depth and time) came to the opposite conclusion^27,28^. This indicates that salinity, unlike other factors, has a greater impact on bacterial than protist community composition.

Apart from changes in community composition (beta diversity), the patterns of species richness (alpha diversity) are of high interest for understanding and monitoring brackish ecosystems. A classical model from the 1930s, Remane’s *Artenminimum* (species-minimum) concept, developed for macrozoobenthos, predicts a minimum in diversity across the salinity gradient at 5-8 PSU, i.e., at the “horohalinicum”^29^. However, studies on fish^30^, and macroalgae^31^ have shown a steady increase in richness with salinity, challenging the generalizability of Remane’s model. For phytoplankton, patterns of both a maximum^32^ and a minimum^33^ of species richness within the mid-range brackish salinity spectrum, as well as an increase along the salinity gradient^34^, have been reported based on microscopic assessments. In contrast, DNA metabarcoding transect surveys of plankton showed no clear patterns for bacteria^14,17,18^ nor for major protist taxa^18^. At the same time, bacterial alpha diversity has been observed to be higher in winter than in summer in the Baltic Sea^17,35^, consistent with patterns observed in marine environments^36–38^. Little is known about the factors driving the seasonal patterns in the Baltic Sea or how the seasonal patterns may confound the alpha diversity patterns along the brackish salinity gradient.

**Fig. 1:**
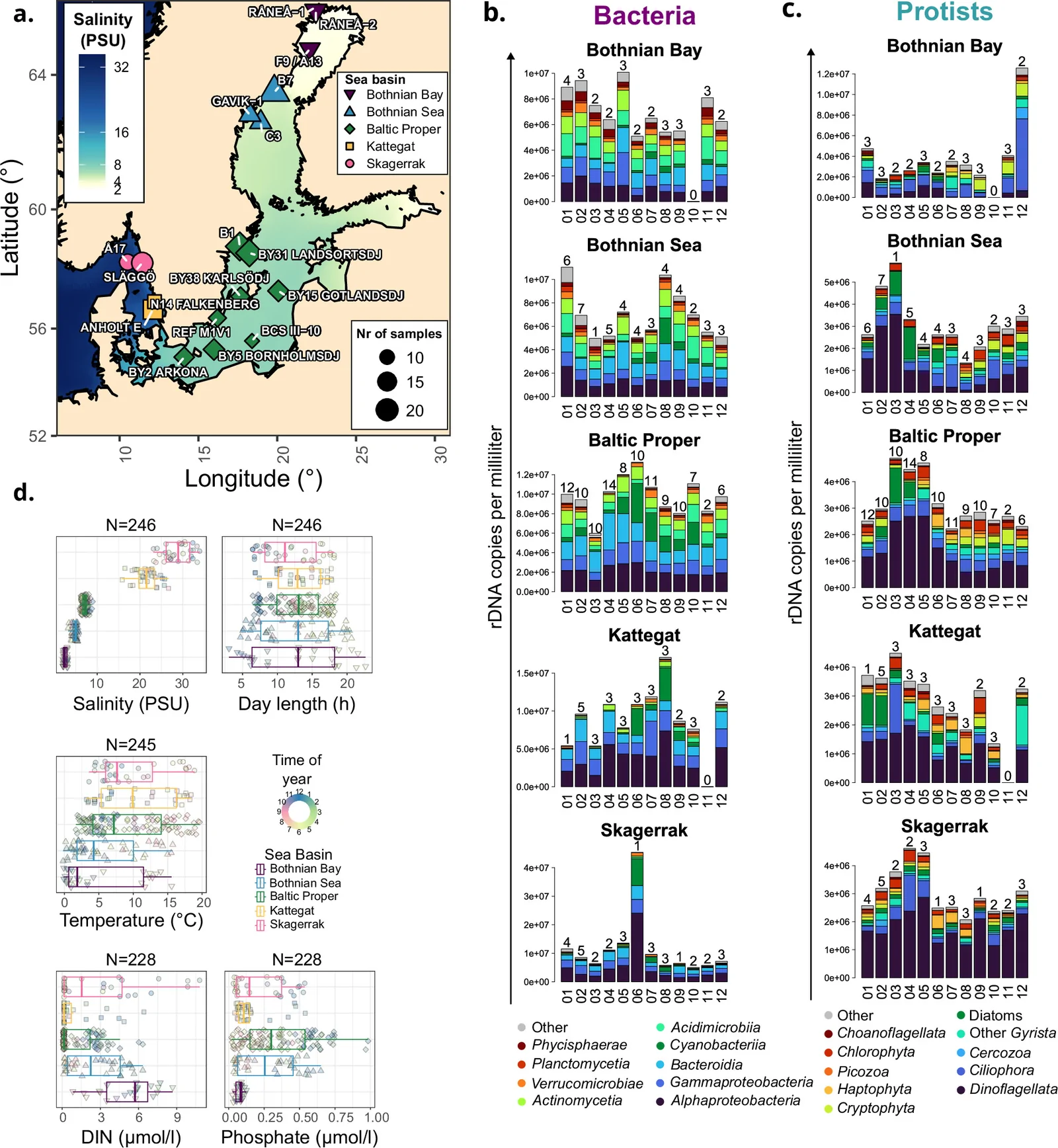
Spatiotemporal changes in abundance of major microbial taxa, 2019-2020. **a** A map of 18 sampling locations labeled by the name of the station and size-coded by the number of samples collected over a period of 13 months (January 2019 to February 2020). The map was made based on Bio-ORACLE^102^ and GSHHG^103^ data, and using the PBS mapping R package^104^. **b**, **c** The numbers of rRNA marker gene copies per liter annotated to (**b**) bacterial classes and (**c**) protist subdivisions averaged over each calendar month and Baltic Sea area basin (as marked in **a**). The values are based on the number of reads relative to spike-in reads and the amount of spike-in added per volume of sample. Only the classes/subdivisions which, on average, corresponded to >1% of reads across all samples are distinguished, and the rest is assigned to the group “Other”. *Gyrista* has been divided into diatoms and other *Gyrista*. Above each bar, the number of samples collected in the respective month and basin is given. Note: Two samples (January in Bothnian Bay and September in Bothnian Sea) were removed for protists due to spike-in detection failure. **d** The distribution of measurements of major physicochemical and geographic parameters for each basin. The lines on the boxplot represent (from the middle) median, 75th percentile, and the whiskers extend to 1.5 times the interquartile range. The number of samples for which the relevant measurement was available is given above each plot. DIN - dissolved inorganic nitrogen.

The Baltic Sea and the adjacent Kattegat-Skagerrak (the Baltic Sea area, Fig. 1a) are among the best-studied brackish waters and coastal areas^39^. Recent formation (8000 years ago^40^), pronounced environmental gradients, and narrow connecting straits make the Baltic Sea a useful model for studying eco-evolutionary processes driven by dispersal and adaptation^41^. In addition, its early exposure to anthropogenic pressures can foreshadow the processes awaiting other coastal regions^42^. The Baltic Sea area also has a long record of microbial research, with the earliest data coming from the 19th century^43^. Regular microscopy-based phytoplankton monitoring has been run by multiple countries in the region for decades^44^ and since the 1980s in Sweden^45^. More recently, the Baltic Sea area was one of the first brackish waters surveyed using DNA metabarcoding^14,18,46^ and metagenomics^12,47^. Nevertheless, most of the unknown diversity in the Baltic Sea area is believed to be microbial^48^.

Here, we present the first in-depth comparative analysis of bacterial and protist diversity across the spatiotemporal scales in the Baltic Sea area and the corresponding environmental gradients. We use a recently published DNA metabarcoding dataset^49^ and a new dataset released here, both originating from samples collected along the Swedish National Marine Monitoring Program. The datasets together span more than two complete calendar years and include 398 samples. We contrast the alpha and beta diversity patterns of bacteria and protists along brackish salinities and other environmental gradients of the Baltic Sea area. We link the differences between the bacterial and protist beta diversity patterns to systematic differences in salinity niche breadths. Finally, we connect our metabarcoding results with publicly available sequences from freshwater and marine samples, showing that distinct distributions of freshwater- and marine-associated taxonomic units underlie the contrasting bacterial and protist alpha diversity patterns across the brackish salinity gradient.

## Results and discussion

### Regionally distinct seasonal dynamics of major bacterial and protist taxa

We first analyzed a 16S and 18S rRNA gene metabarcoding dataset recently published by us, coming from over a year of biweekly sampling at multiple locations covering broad environmental gradients^49^ (Fig. 1a, d). Amplicon sequence variants (ASVs) were clustered by simultaneous genetic and distribution similarity, using distribution-based operational taxonomic unit-calling^50^ (we call these dbOTUs). We thus obtained 16,295 bacterial and 3935 protist dbOTUs. More than half of the dbOTUs were annotated to at least family level for both bacteria and protists (Supplementary Fig. 1c, d). Moreover, more than half of the reads represent dbOTUs annotated to at least genus or family level for bacteria and protists, respectively (Supplementary Fig. 1e, f). Because the samples also contained a known amount of synthetic spike-in DNA amplifiable by the metabarcoding primers, absolute abundances of individual dbOTUs could be estimated.

The major bacterial (Fig. 1b) and protist (Fig. 1c) taxa displayed distinct seasonal abundance dynamics. Both their abundances and seasonal patterns differed between the Kattegat/Skagerrak, and the three basins of the Baltic Sea included in this study (Baltic Proper, Bothnian Sea, and Bothnian Bay). Note that the abundances are given in rRNA gene copies, and as protists often have much higher rRNA gene copy numbers than bacteria^51^, they appear similarly abundant despite being less numerous in reality. Indeed, 18S rRNA gene count-derived abundances of phytoplankton classes have been shown to correlate better with biomass than cell counts^52^, and protists have higher biomass per cell and their total biomass in the Baltic Sea water column is comparable to bacteria^53^.

We observed diatoms peaking and dinoflagellates starting to peak in abundance in March in the Baltic Proper (Fig. 1c), consistent with microscopy-based observations^54,55^. However, the dynamics and abundance of dinoflagellates and diatoms differed vastly between basins. Still, in all cases except for the Bothnian Bay, those groups increased in abundance in the first half of the year. Cyanobacteria increased in abundance as early as May in the Baltic Proper (Fig. 1b), as observed using microscopic methods^55^. The increase in cyanobacterial abundance was smaller and peaked later, moving northwards within the Baltic Sea. *Haptophyta* generally displayed increased abundance between May and August. In the Kattegat-Skagerrak, there was an additional dinoflagellate peak in September, corresponding to autumn blooms. *Cryptophyta* were abundant in the Baltic Sea, with increased abundance in the second half of the year.

We also observed geographic differences in abundance and seasonal dynamics of major heterotrophic bacteria. *Bacteroidia* (Fig. 1b) peaked in abundance in May across the Baltic Sea, consistent with previous reports^35,53^, but displayed less clear seasonal dynamics in the Skagerrak and the Kattegat. *Actinomycetia* and *Acidimicrobiia* were abundant in the Baltic Sea but not in the Kattegat-Skagerrak, consistent with *Actinobacteria* abundance patterns from transect-based studies^14,18,47^. Additionally, *Acidimicrobiia* decreased in abundance between May and July, a dynamic that was more pronounced moving southwards in the Baltic Sea. Finally, contrary to previous results from a near-coast station^35,53^, *Alphaproteobacteria*, and *Gammaproteobacteria* comprised a stable proportion of the whole community throughout the year in all basins but the Kattegat (Supplementary Fig. 2a). Nevertheless, consistent with metabarcoding^14^ and metagenomic transects^47^, *Alphaproteobacteria* and *Gammaproteobacteria* were more abundant in the Kattegat-Skagerrak than in the Baltic Sea.

Compared to relative abundance-based analysis, spike-in correction added ecologically relevant information. For example, the increase in dinoflagellate abundance in the Baltic Proper was observed in March with the spike-in approach (Fig. 1c), consistent with a microscopy-based report for the same year^55^. Meanwhile, their relative abundance increased only in April (Supplementary Fig. 2b), potentially being masked by a simultaneous increase in diatom abundance in March. Nevertheless, the spike-in approach appeared to generate substantial noise, best exemplified by the outlier results for total rDNA counts in some situations when the results were averaged across few samples (Fig. 1b, c), and relative abundances better captured gradual succession patterns, especially for bacteria (Supplementary Fig. 2a). Thus, spike-in correction is useful in cases where absolute abundances are crucial, e.g., bloom detection, but improvements are needed to reduce noise.

### Salinity has a dominant impact on bacterial but not protist community composition

The dissimilarities of bacterial and protist communities between the same pairs of samples were correlated (R^2^ = 0.58). However, the protist communities were generally less similar to each other than bacterial ones (Supplementary Fig. 3a), as has been reported for picoeukaryotes and bacteria in ocean surface waters^56^.

Principal Coordinate Analysis (PCoA) showed stronger geographic than seasonal structuring of bacterial communities, while for protists the effects of geography and season were comparable (Supplementary Fig. 4). To disentangle the environmental factors driving those structuring effects, we performed distance-based Redundancy Analysis (dbRDA). We focused on five fundamental environmental factors: salinity, temperature, day length (i.e., daytime length), dissolved inorganic nitrogen (DIN), and phosphate. These factors did not strongly correlate with each other (*R*^2^ < 0.4, Supplementary Fig. 5) and were measured for the majority of the samples. While we do not expect day length to have strong direct impacts on most planktonic microbes, it has been repeatedly correlated with seasonal changes in bacterial alpha diversity^36,38^. We thus use it as a hypothetical proxy for commonly observed multifactorial seasonal changes.

**Fig. 2:**
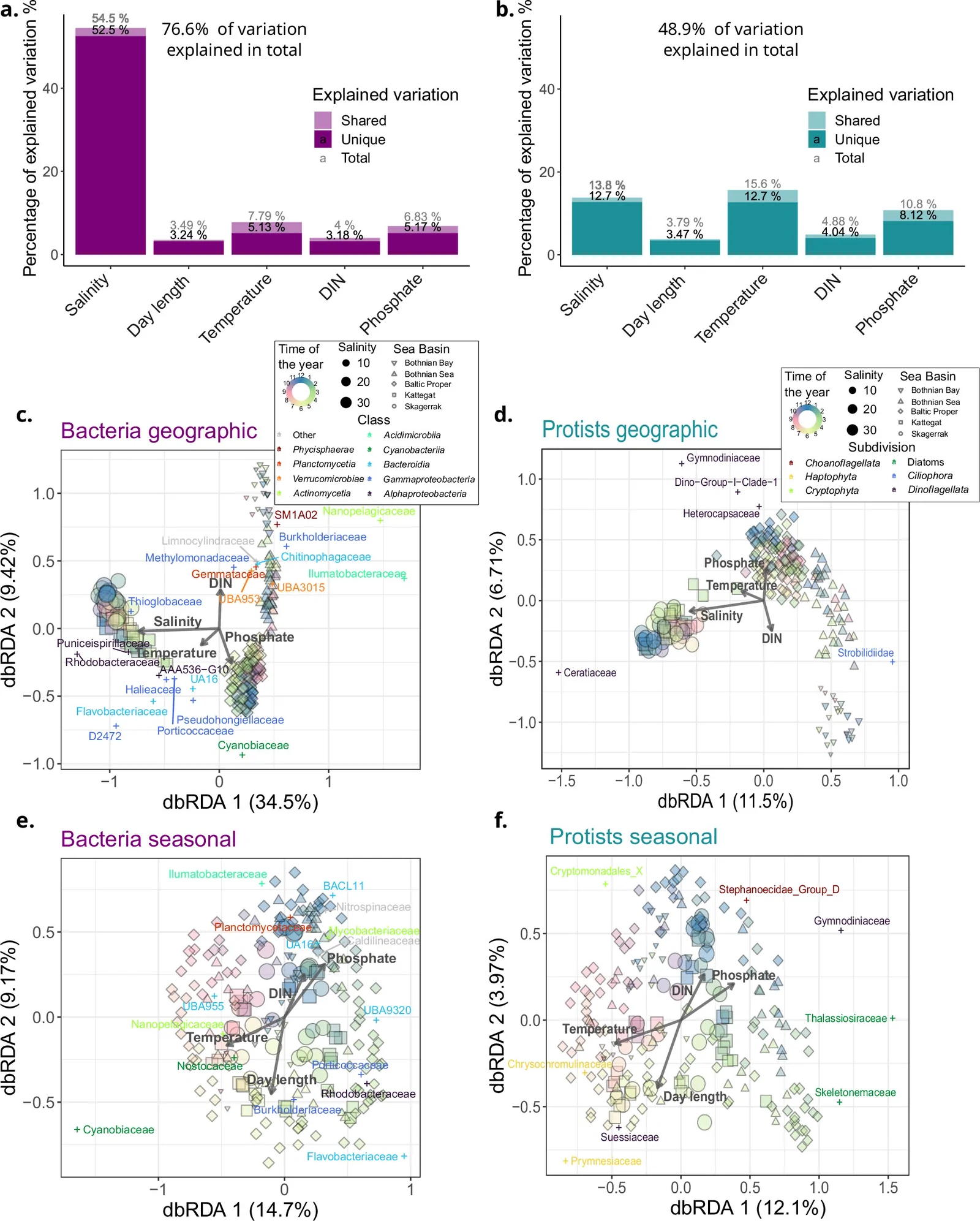
Environmental factors explaining geographic and seasonal community structuring and the families of bacteria and protists most strongly correlated with the beta diversity patterns. **a**, **b** Variance partitioning of bacterial (**a**) and protist (**b**) beta diversity, based on Bray-Curtis distances and distance-based Redundancy Analysis (dbRDA). **c**, **d** Partial dbRDA conditioned on seasonality (sine and cosine of the day of the year), highlighting bacterial (**c**) and protist (**d**) geographic community structuring. **e**, **f** Partial dbRDA conditioned on salinity and latitude, highlighting bacterial (**e**) and protist (**f**) seasonal community structuring. **c–f** Weighted averages of families significantly deviating from the general distribution of families across the axes (Bonferroni-corrected *P* < 0.05) were placed on the plot, with values rescaled by a factor of 0.5. The families were color-coded according to classes/subdivisions (low abundance classes are represented by “Other”). *Gyrista* was divided into diatoms and other *Gyrista*. Percentages of variation explained by each dbRDA component are given in brackets by the axes labels. The same number of samples (*N* = 228) was used for all the panels. DIN - dissolved inorganic nitrogen.

The dbRDA-based variation partitioning showed that salinity could explain around half of the variation in bacterial community composition (Fig. 2a). The effect of salinity on protist community composition was much smaller ( ~ 13%), while temperature and nutrients were of greater relative importance than for bacteria (Fig. 2b). These results contradict the proposed rule that the distribution of smaller organisms is less limited by environmental factors than that of larger organisms^57^, and in particular that bacteria are less environmentally filtered than protists^27^. Rather, our results are consistent with a previous report of a stronger selection of bacterial than protist communities across a salinity gradient^16,26^. However, we do not find support for the generalization of the stronger selection by other environmental factors^26^.

Nevertheless, for both bacterial and protist communities, there was a pronounced shift coinciding with the salinity divide between the Baltic Sea and the Kattegat-Skagerrak and a more gradual change along the Baltic Sea salinity gradient (Fig. 2c, d). We identified microbial families whose abundance most strongly followed these changes in dbOTU-based beta diversity (Fig. 2c, d). For example, typically freshwater *Nanopelagicaceae* (acI *Actinobacteria*)^58^ and *Limnocylindraceae* (*Chloroflexi*)^59^ were associated with the lowest salinity samples, while commonly marine *Rhodobacteraceae*^60^ and *Thioglobaceae*^61^ were at the other end of the salinity spectrum (Fig. 2c). Regardless, the observed geographic patterns of beta diversity were also surely influenced by other abiotic and biotic factors such as climate, loads and composition of terrestrially derived organic matter, and particulate nutrient concentrations.

Both bacterial and protist communities underwent gradual community composition changes throughout the year in all the analyzed basins of the Baltic Sea area (Fig. 2e, f, Supplementary Fig. 6). Diverse groups significantly contributed to the seasonal patterns in beta diversity, ranging from known seasonally blooming diatoms *Thalassiosiraceae* and *Skeletonemaceae*^62^ to multiple uncharacterized *Bacteroides* families. In all the basins, picocyanobacteria (*Cyanobiaceae*) were associated with high temperatures, while BACL11 (*Bacterioides*) were typical of winter conditions (Fig. 2e, Supplementary Fig. S6a).

### Interannual recurrence of spatiotemporal patterns of microbial community composition

**Fig. 3:**
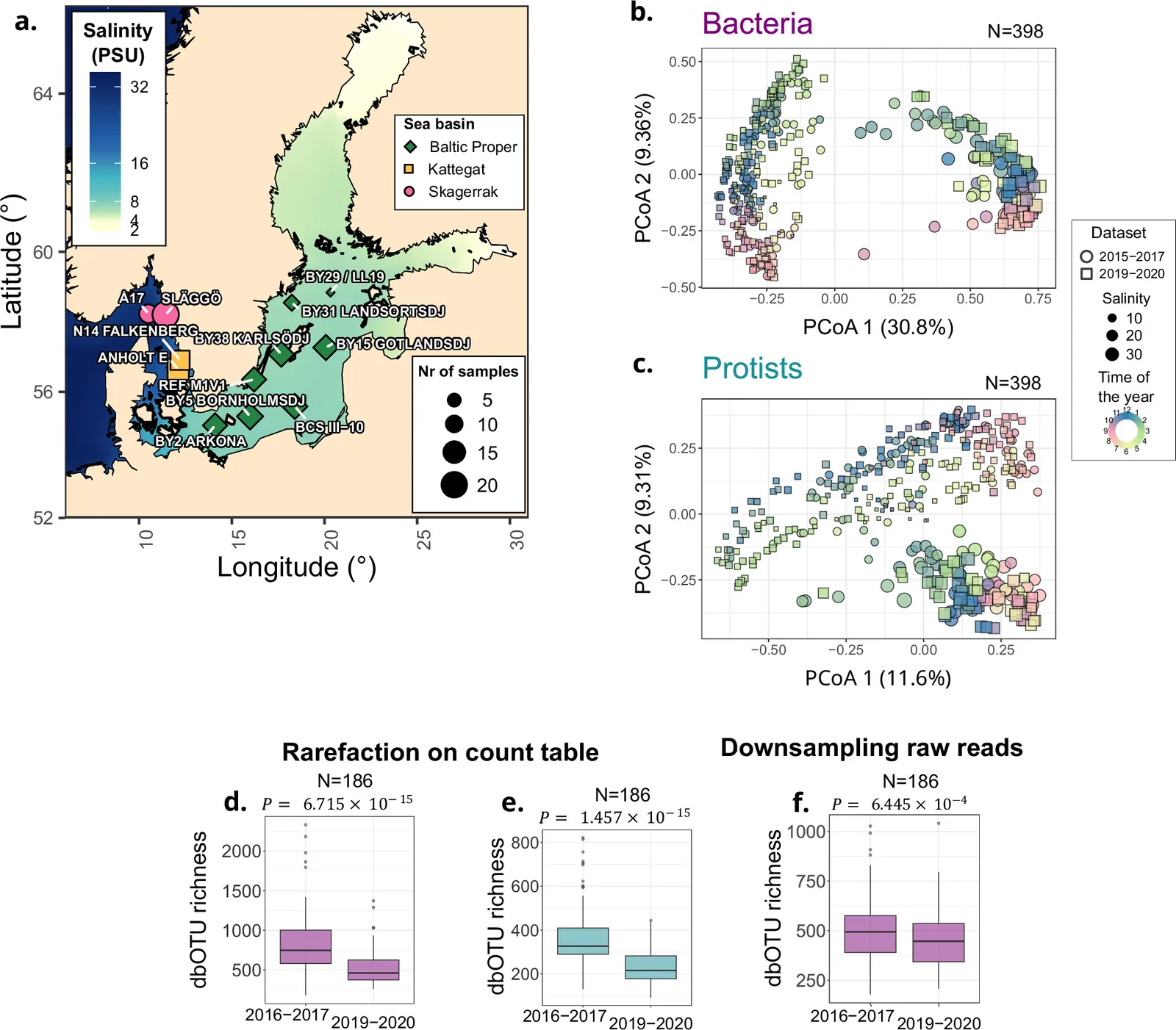
Bacterial and protist diversity across datasets collected in 2015–2017 and in 2019–2020. **a** A map of 12 sampling locations labeled by the number of samples collected between August 2015 and March 2017 (the 2015-2017 dataset), with all the 2015 samples coming from station Släggö in Skagerrak. The map was made based on Bio-ORACLE^102^ and GSHHG^103^ data, and using the PBS mapping R package^104^. **b**, **c** PCoA based on Bray-Curtis dissimilarities of bacterial (**b**) and protist (**c**) communities across the 2015–2017 and 2019–2020 datasets. **d**, **e** dbOTU richness of bacteria (**d**) and protists (**e**) in the two datasets. Samples of both datasets were rarefied to the same number of reads. Only dbOTU richness values for stations present in both datasets have been used. Moreover, samples were chosen in pairs, from the same station and the same month of the year but different datasets. Sample pairs with the lowest distance in terms of the day in a calendar year were chosen. *P-*values obtained using the pairwise, two-sided Wilcoxon (signed-rank) test are given above each plot. **f** Same as (**d**) but based on richness values obtained by downsampling raw reads. The differences in the number of samples come from the availability of physicochemical parameters or (**d**–**f**) the interannual data balancing approach. The number of samples (N) used to create the plots is given above each panel. DIN - dissolved inorganic nitrogen, dbOTU - distribution-based operational taxonomic unit.

We further assessed whether the identified beta diversity patterns could be reproduced in an independent dataset from a different timeframe. We thus performed metabarcoding on stored DNA extracted from samples collected between 2015 and 2017 (Fig. 3a, Supplementary Fig. S7a, see Methods for details).

Overall, the community composition in the two datasets differed minimally, following the spatial and seasonal environmental gradients analogously (Fig. 3b, c). This apparent spatiotemporal recurrence of community composition is consistent with previous observations from the Baltic Sea^53,63^ and other waters^28^. Moreover, the similarity of communities between the two datasets shows that the technical differences (see Methods for details) had little impact on the observed general community composition (Supplementary Fig. S7b-c). However, the datasets were separated by only two years, and the interannual stability is unlikely to hold in the longer timeframe as global change progresses. For example, there is strong evidence for a long-term change in diatom-dinoflagellate ratio during spring bloom in the Baltic Sea^62^.

In contrast to community composition, alpha diversity differed significantly (*P* < 10^-14^ in both cases) for both bacteria and protists (Fig. 3d, e). We doubt the increased richness in the 2015-2017 dataset, amounting to hundreds of dbOTUs on average, represented an actual biological change. Indeed, switching to downsampling raw reads instead of downsampling dbOTU counts removed nearly all of the difference in dbOTU richness between the 2015-2017 and 2019-2020 datasets (Fig. 3f). We also checked that downsampling raw reads consistently resulted in lower richness than downsampling the ASV counts (Supplementary Fig. S8). This is consistent with a previous report showing that downsampling before obtaining ASVs better removes sequencing-depth biases^64^. However, a small but significant difference in richness between datasets remained after downsampling reads (Fig. 3f), indicating that other technical or biological factors also contributed to the difference in alpha diversity between the datasets.

### Seasonal vertical mixing brings deep-water bacteria to the surface and increases bacterial alpha diversity

**Fig. 4:**
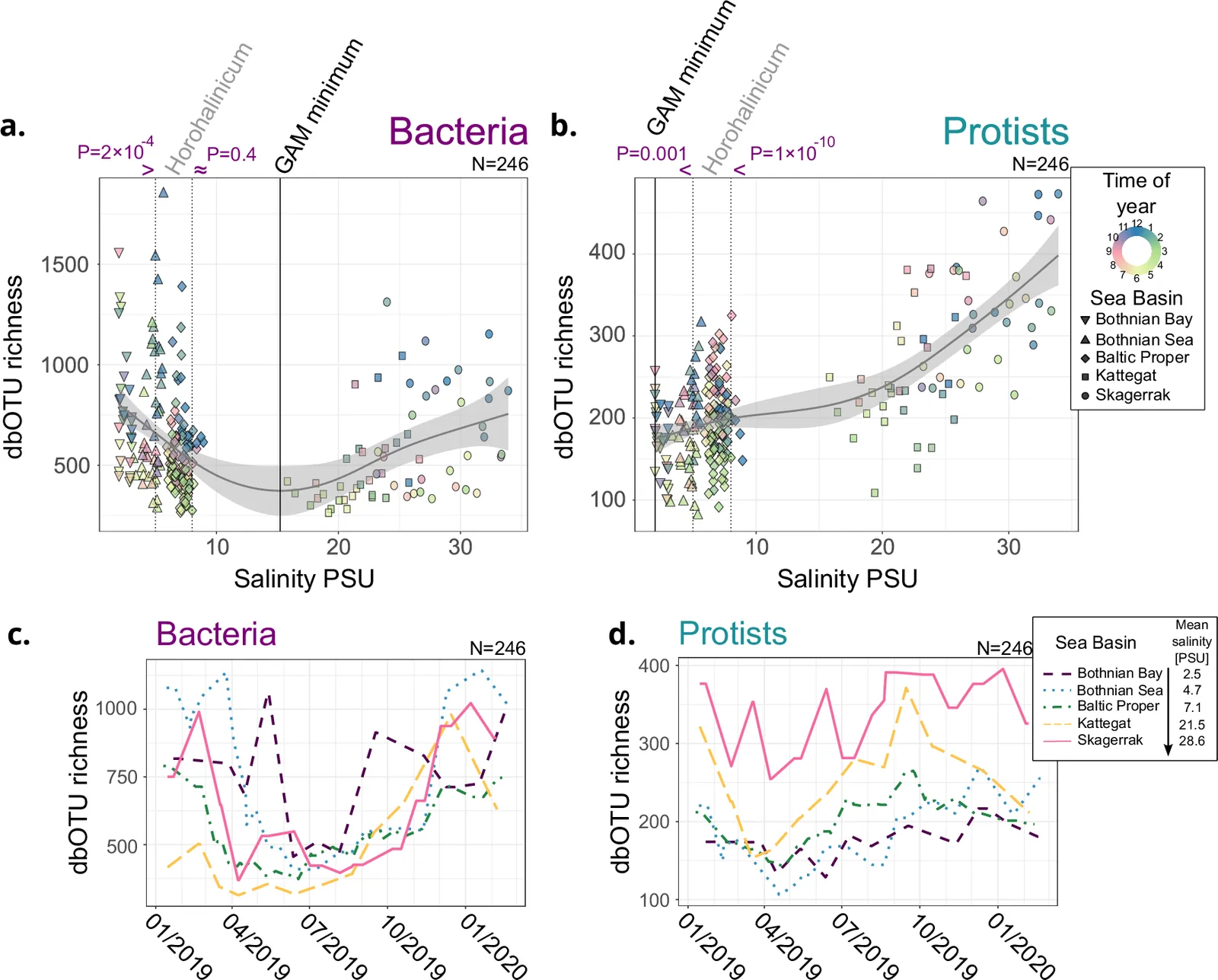
Bacterial and protist richness. Mean number of bacterial (**a**) and protist (**b**) dbOTUs found across 100 rarefaction iterations (dbOTU richness). The fit corresponds to smoothened generalized additive models (GAMs). The dotted vertical lines correspond to boundaries of horohalinicum (5-8 PSU). The colored (in)equality signs over the dotted lines mark the significant differences in dbOTU richness between horohalinicum and lower/higher salinities, and the *P*-values from two-sided Wilcoxon rank-sum tests are written above. The solid vertical lines mark salinities corresponding to minimum richness in the GAMs. The same legend applies to (**a**) and (**b**). The solid gray line represents the richness values predicted by the GAMs, and the gray area corresponds to ± standard error. **c**, **d** Rolling means of bacterial (**c**) and protist (**d**) richness for the distinguished Baltic Sea area basins. The richness values were averaged for measurements 2 weeks before and after each date. The same legend applies to (**c**) and (**d**). The number of samples (N) used to create the plots is given above each panel. The differences in the number of samples come from the availability of physicochemical parameters.

Next, we analyzed the changes in alpha diversity along the salinity gradient. We applied the same statistical methods as were used in previous studies to argue for or against maximum^18,32^ and minimum^33^ protist diversity in mid-range brackish salinities (Fig. 4a, b). The horohalinicum, i.e., midrange salinities (5-8 PSU), displayed lower bacterial richness than in less (but not more) saline waters (Fig. 4a). The minimum in bacterial richness was at 15 PSU according to the generalized additive models. However, the minimum estimation was likely confounded by seasonal changes in salinity within Kattegat, as there was clear seasonal variation in bacterial richness overshadowing the geographic differences (Fig. 4a, c).

Across geographic regions, bacterial alpha diversity rapidly declined in spring and increased again in autumn (Fig. 4c). Out of the assessed environmental factors, DIN explained the largest part of the variation (Supplementary Fig. S9a).

Spring depletion of nitrogen in the Baltic Sea is closely connected to the emergence of the summer thermocline and thus reduced vertical mixing^65^. Winter vertical mixing has been linked to elevated bacterial alpha diversity and similarity in cytometric properties across the water column in a marine time series^66^. Additionally, some members of typically deepwater clades are rare taxa in surface waters with abundance maxima in winter^67^. The influx of deepwater bacteria has also been suggested to explain the higher diversity of bacteria in winter than in summer in the Baltic Sea, with winter communities being less structured by depth^17^. However, it remains unclear whether the influx of deepwater taxa or changes in environmental conditions concurrent with vertical mixing underlie the diversity pattern.

Therefore, we connected our results with a previously published metabarcoding dataset spanning different depths across a winter and a summer transect in the Baltic Sea^17^, from here on referred to as the “transect-based dataset”. We matched the sequences representative of the operational taxonomic units (OTUs) from the transect-based dataset to ASVs representative of our dbOTUs (see Methods for details).

**Fig. 5:**
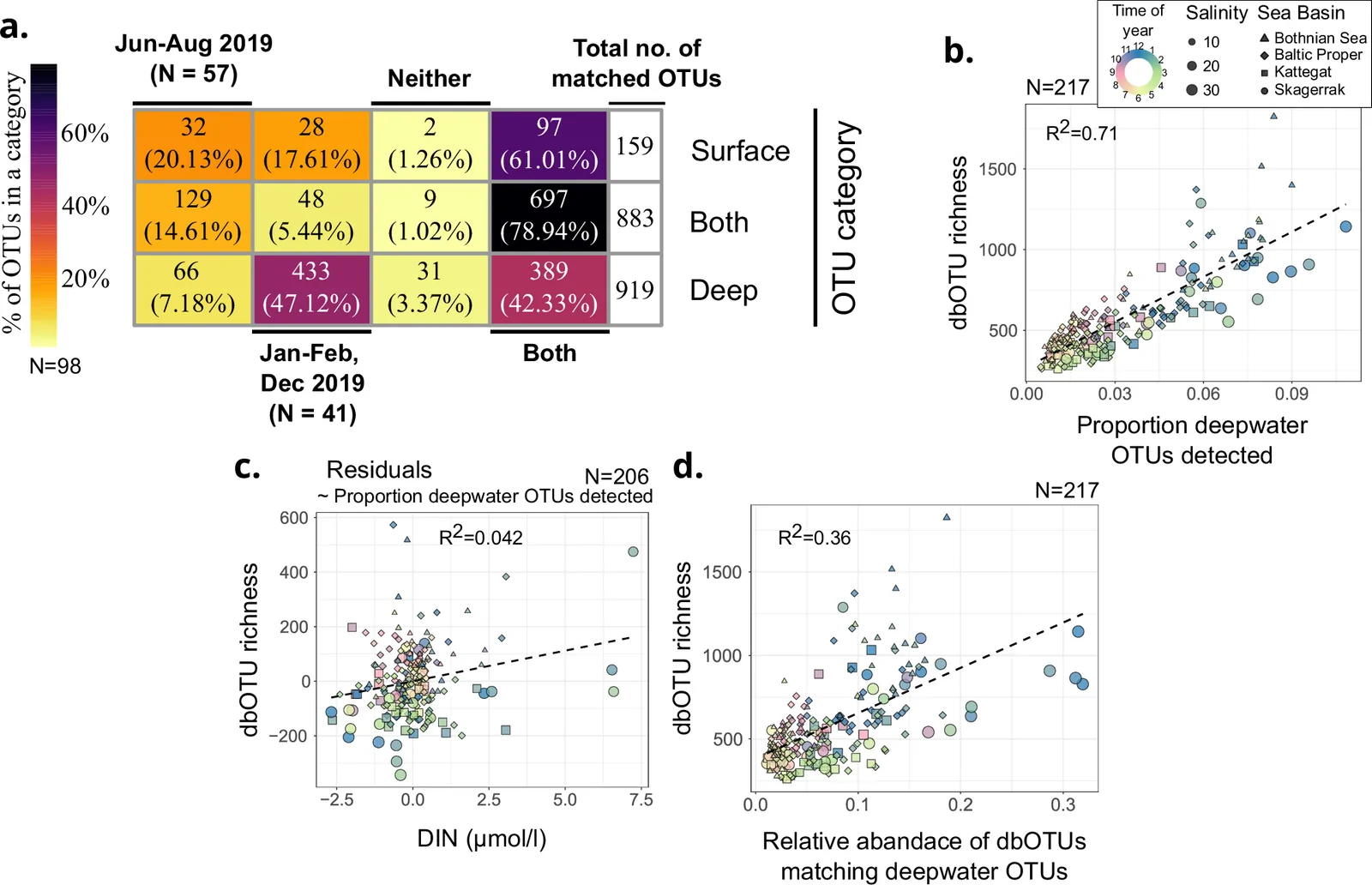
Bacterial alpha diversity and the proportion of deepwater OTUs detected. **a** Number of OTUs from a previous, transect-based study^17^ matched to dbOTUs found in winter and summer samples or in neither or both seasons. The OTUs are categorized by being found exclusively in deep water ( > 10 m depth) samples, exclusively in surface waters, or in both, in the original, transect-based study. Only OTUs matching any dbOTUs from this study are counted. *P*-values for the proportion of OTUs detected in summer being equal were 10^-73^ for deep, 10^-8^ for both, and 0.26 for surface. **b** Proportion of deepwater OTUs (including ones without a match in the 2019–2020 dataset) having a match to a dbOTU from a sample vs. bacterial dbOTU richness. **c** The residuals of DIN vs. dbOTU richness from models correlating the factors to the proportion of deepwater OTUs detected, corresponding to partitioning out the unvisualized parameter. **d** Relative abundance of all dbOTUs matching a deepwater OTU vs bacterial dbOTU richness. Bothnian Bay was excluded from the plots since there were no deepwater samples taken in this region in the transect-based study^17^. The number of samples (N) used to create each panel is given in each panel. The differences in the number of samples come from the availability of physicochemical parameters and data division strategies (**a**). The same set of samples from the transect-based study^17^ (N = 106) was used for all the panels (samples not shown, see Methods for details). The same legend applies to (**b**)–(**d**). DIN dissolved inorganic nitrogen.

We further chose from the transect-based dataset only the summer (July) samples. Among those, we categorized OTUs as found only in deep ( > 10 m depth) or in surface ( ≤ 10 m depth) waters, or in both. The surface mixed layer in summer in the Baltic Sea is usually located above 10 m depth^65^. We assessed how many OTUs from each category matched the dbOTUs from winter (December–February) and summer (June–August) months (Fig. 5a). Among the deepwater OTUs, significantly more (*P* < 10^-73^) were found in our surface water samples in winter than in summer, despite originating from summer samples.

The proportion of detected deepwater OTUs in a sample strongly correlated with dbOTU richness (Fig. 5b). In contrast, the proportion of detected non-deepwater-specific OTUs (‘surface’ and ‘both’ from Fig. 5a) showed a weaker correlation with richness (Supplementary Fig. S9c), and did not follow the proportion of deepwater OTUs matched (Supplementary Fig. S9d). Thus, the correlation between the proportion of deepwater OTUs and richness did not originate from detection biases. Moreover, after partitioning out the correlations with deepwater OTUs, DIN no longer correlated with dbOTU richness (Fig. 5c). This relation was not bilateral; the proportion of deepwater OTUs still correlated with dbOTU richness after partitioning out DIN (Supplementary Fig. S9e). Therefore, seasonal convective water mixing increases winter alpha diversity by bringing deepwater bacteria to the surface and increasing the observed richness, consistent with previous reports^17,36,38,66,67^.

In winter, deepwater taxa not only increased alpha diversity but also comprised a substantial part of the surface water bacterial communities, up to >30% in Skagerrak (Fig. 5e). With cumulative relative abundance commonly above 10% in winter, the deepwater taxa likely impact the surface water ecosystem, if not through metabolic activity, at least by providing alternative food sources for heterotrophs. A metatranscriptomic approach would be needed to determine whether the deepwater bacteria are active in surface waters in winter.

Ultimately, the higher mean bacterial richness in the low salinity area of the Bothnian Sea and the Bothnian Bay (Fig. 4a) can be explained by differences in seasonal dynamics, i.e., a longer period of vertical mixing due to colder climates^9^. In summer, when mixing ceased, richness dropped to similar levels across basins (Fig. 4c), consistent with previous findings reporting no salinity-dependent pattern of bacterial alpha diversity^14,17,18^.

### Higher protist alpha diversity in near-marine salinities

In contrast to the situation for bacteria, protist richness displayed a clear salinity-dependent pattern, monotonically increasing with salinity (Fig. 4b; linear fit yields R^2^ = 0.44). This is consistent with a recent microscopy-based study^34^, while it contrasts with reports of other patterns^18,32,33^. However, unlike in the microscopy study by Olofsson et al.^34^, richness in the near-marine Kattegat-Skagerrak was higher than in the Baltic Sea across the year (Fig. 4d).

### Protist dbOTUs have broader salinity niches than bacterial dbOTUs

Salinity had a stronger impact on bacterial beta diversity (Fig. 2) but protist alpha diversity (Fig. 4). Alpha diversity is unaffected if one taxonomic unit is replaced with another, and a taxon’s impact on beta diversity is proportional to its abundance. Thus, based on the diversity patterns, it is unclear whether all bacteria or only the abundant ones have narrower salinity niches than their protist counterparts. We therefore proceeded to more directly analyze the realized salinity niches of bacterial and protist dbOTUs.

**Fig. 6:**
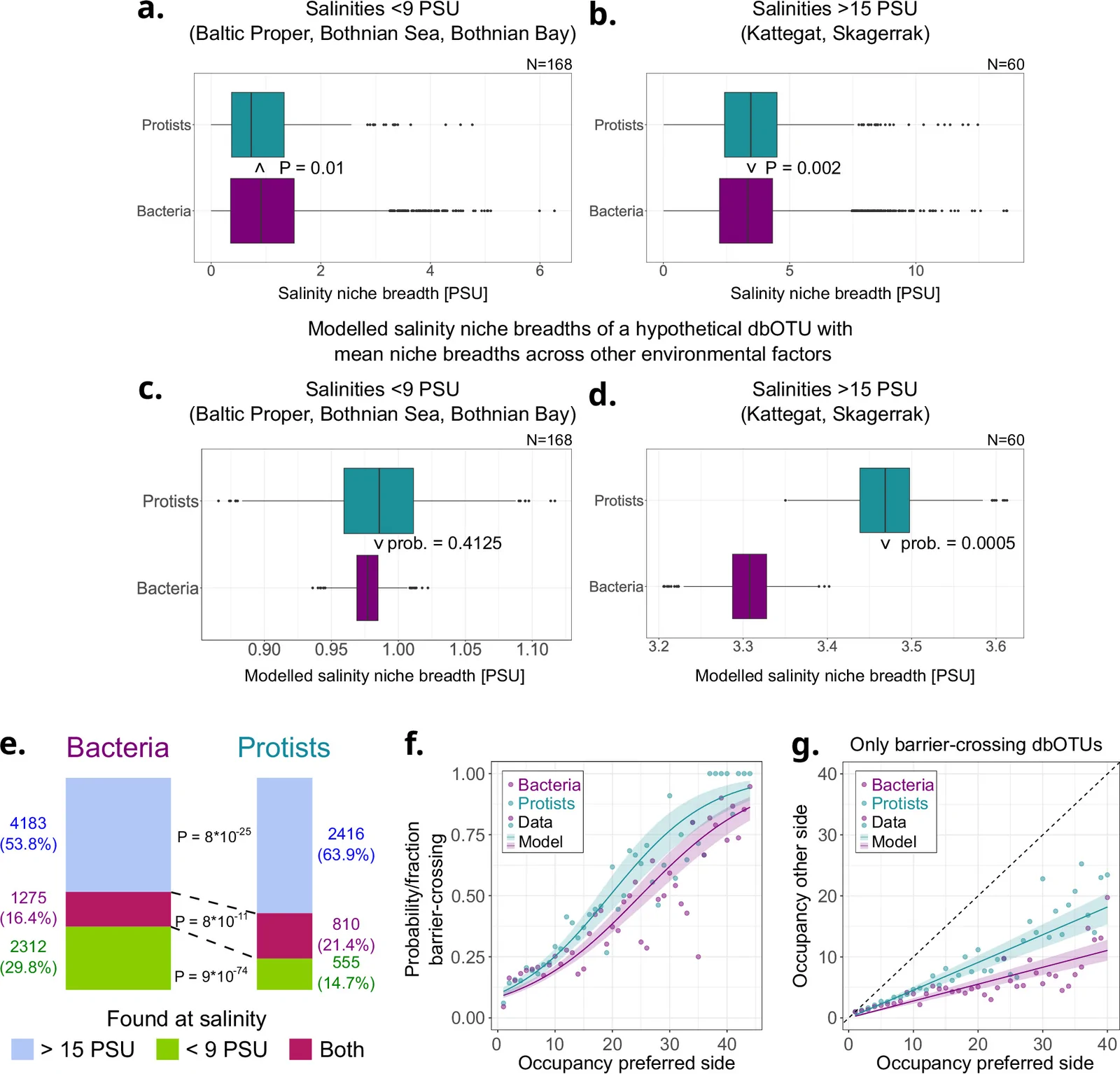
Salinity niche breadths and proportions of salinity barrier-crossing bacteria and protists. **a**, **b** The estimated salinity niche breadths of dbOTUs found only in salinities below 9 PSU (**a**) or above 15 PSU (**b**). **c**, **d** Results of Bayesian modeling. Predicted salinity niches for a hypothetical dbOTU with average temperature, DIN, phosphate, and day length niche breadths, depending on whether it would be a bacterium or a protist. The modeling has been done using dbOTUs found only in salinities below 9 PSU (**c**) or above 15 PSU (**d**). In (**a**)–(**d**), N represents the number of samples with salinities below 9 PSU or above 15 PSU. The sign between boxplots indicates which group had broader salinity niches, and *P*-values (**a**, **b**) or probabilities of obtaining an opposite trend in the Bayesian model (**c**, **d**) are given. The lines on the boxplot represent (from the middle) median, 75th percentile, and the whiskers extend to 1.5 times the interquartile range. The significance values in (**a**, **b**) come from two-sided Wilcoxon rank-sum test and the probabilites in (**c**, **d**) from the results of the Bayesian modeling (proportion of MCMC iterations yeilding a difference with a different direction to the difference between the medians). **e** The number and proportion of bacterial and protist dbOTUs found in high ( > 15 PSU) and low ( < 9 PSU) brackish salinities, and across the salinity barrier. The significance values comes from two-sided Fisher’s exact test. **f**, **g** Results from Bayesian modeling. **f** The proportion of salinity barrier-crossing dbOTUs (found on both sides of the Danish Straits; points) and the modeled probabilities of a dbOTU with a given occupancy crossing the salinity barrier. **g** The occupancy on the less occupied side of the salinity barrier as a function of the occupancy on the more occupied side. In (**f**) and (**g**), the points correspond to the means of the observed values. The lines correspond to the values predicted from the model with 95% confidence intervals in shaded colors. In (**g**), the dashed black line is the identity line. For (**e**)–(**g**), only the two stations with the highest salinities (21-34 PSU), and two stations with salinities around 6-7 PSU were included. The same number of samples (n = 22) were included for each station, as close in time as possible between stations (see Methods for details). “Occupancy preferred side” and “occupancy other side” refer to the occupancy at the side of the salinity barrier with higher and lower occupancy, respectively.

First, we estimated the salinity niches of dbOTUs found only in salinities higher ( > 15 PSU) or lower ( < 9 PSU) than in the Danish Straits (Fig. 6a-d). We thus based each analysis on a unimodal distribution of salinity values. Among the Baltic Sea dbOTUs (lower salinities), bacterial ones had broader salinity niches (Fig. 6a), while in Kattegat/Skagerrak (higher salinities), it was protist dbOTUs that were usually found across a broader range of salinities (Fig. 6b). However, the differences between the groups were small compared to the spread of salinity niche breadths within the groups. It is expected that microbial niches are limited by multiple factors, which leads to salinity-independent limitations in dbOTUs detection range. To correct for the salinity-independent effects, we built a Bayesian model of bacterial and protist salinity niche breadths based on temperature, DIN, phosphate, and day length niche breadths. We then predicted and compared salinity niche breadths for hypothetical bacterial and protist dbOTUs with the same (mean) niche breadths across other factors (Fig. 6c, d). After correcting for other factors, the bacterial salinity niches in the Baltic Sea were not broader than for protists (Fig. 6c), while the trend towards broader salinity niches of protists than bacteria in Kattegat/Skagerrak became even stronger (Fig. 6d).

To estimate how commonly bacteria and protists have salinity niches spanning across the Danish Straits, we analyzed the proportions of dbOTUs found on both sides of the salinity barrier. To limit the effect of other factors on the analysis, we selected two stations on each side of the salinity barrier, keeping only near-marine stations (in Skagerrak) and stations in the Baltic Proper considerably far from the barrier itself, to lower the risk of detecting temporary barrier-crossing plankton due to storm events, transport with water currents, or other causes. We combined the 2019-2020 and 2015-2017 datasets and used samples as close in time as possible, resulting in 88 samples in total (22 per station, see Methods for details). A significantly higher proportion of protist dbOTUs occurred across the salinity barrier (Fig. 6e).

However, although we rarefied the samples to the same read depth, this simple analysis could be confounded by detection limits. Indeed, even on the preferred side of the salinity barrier, protist dbOTUs were on average found in a larger number of samples (Supplementary Fig. S10). We thus modeled the probability a dbOTU was found on both sides of the salinity barrier as a function of occupancy (i.e., the number of samples in which a dbOTU was found). This Bayesian model showed that protists were more likely to cross the barrier than bacteria across the occupancy values (Fig. 6f). For all the occupancy values, the probability of protists being more likely to cross the barrier was >99%.

Furthermore, among the dbOTUs that crossed the salinity barrier, protists tended to be present in more samples at the less-preferred side of the barrier (Fig. 6g). Thus, the barrier-crossing dbOTUs were more likely to grow on both sides of the barrier, as opposed to their genetic material being transported with currents.

Overall, protist dbOTUs had wider salinity niches than their bacterial counterparts, though this effect was not observed at the lower end of the brackish salinity gradient (Fig. 6). This suggests that the diversity patterns we observed present a general trend of different breadth of realized niches with respect to salinity across ecologically diverse bacteria and protists. Nevertheless, taxa or ecological groups that deviate from this trend could surely be found.

### Among protists, but not bacteria, marine-associated lineages outnumber freshwater-associated ones across most of the salinity gradient

Similarly to our observations on protists, fish^30^ and macroalgal^31^ alpha diversity increases with salinity in the Baltic Sea area. The Baltic fauna and macroalgae stem mainly from a limited number of local adaptations to brackish conditions following the last glaciation period, with marine lineages outnumbering freshwater ones^31,41,48^. In contrast, for Baltic Sea bacteria, there is evidence against widespread occurrence of such local adaptation. Instead, the niches appear to be filled through colonization by a global brackish microbiota, consisting of species that are distinct from but often related to freshwater and marine lineages^8^. Consistently, in the current (Fig. 4a, c) and previous^14,17,18^ studies, bacterial alpha diversity remained relatively stable across the brackish salinity gradient. A metabarcoding-based analysis by Herlemann et al. showed that in the Baltic Sea, freshwater-associated bacterial taxa are gradually replaced by marine-associated ones, keeping alpha diversity constant^14^.

To test whether the difference in alpha diversity patterns between bacteria and protists can be explained by the distribution of marine- and freshwater-associated lineages, we matched our dbOTUs to surface water ( < 10 m depth) freshwater and marine ( > 34 PSU) ASVs from the 16S rRNA gene database MiCoDa^68^ and the 18S rRNA gene database MetaPR^2^^69^. The 16S rRNA gene ASVs in MiCoDa are 90bp-long and we trimmed the MetaPR^2^ ASVs to the same length for comparability (see Methods for details).

**Fig. 7:**
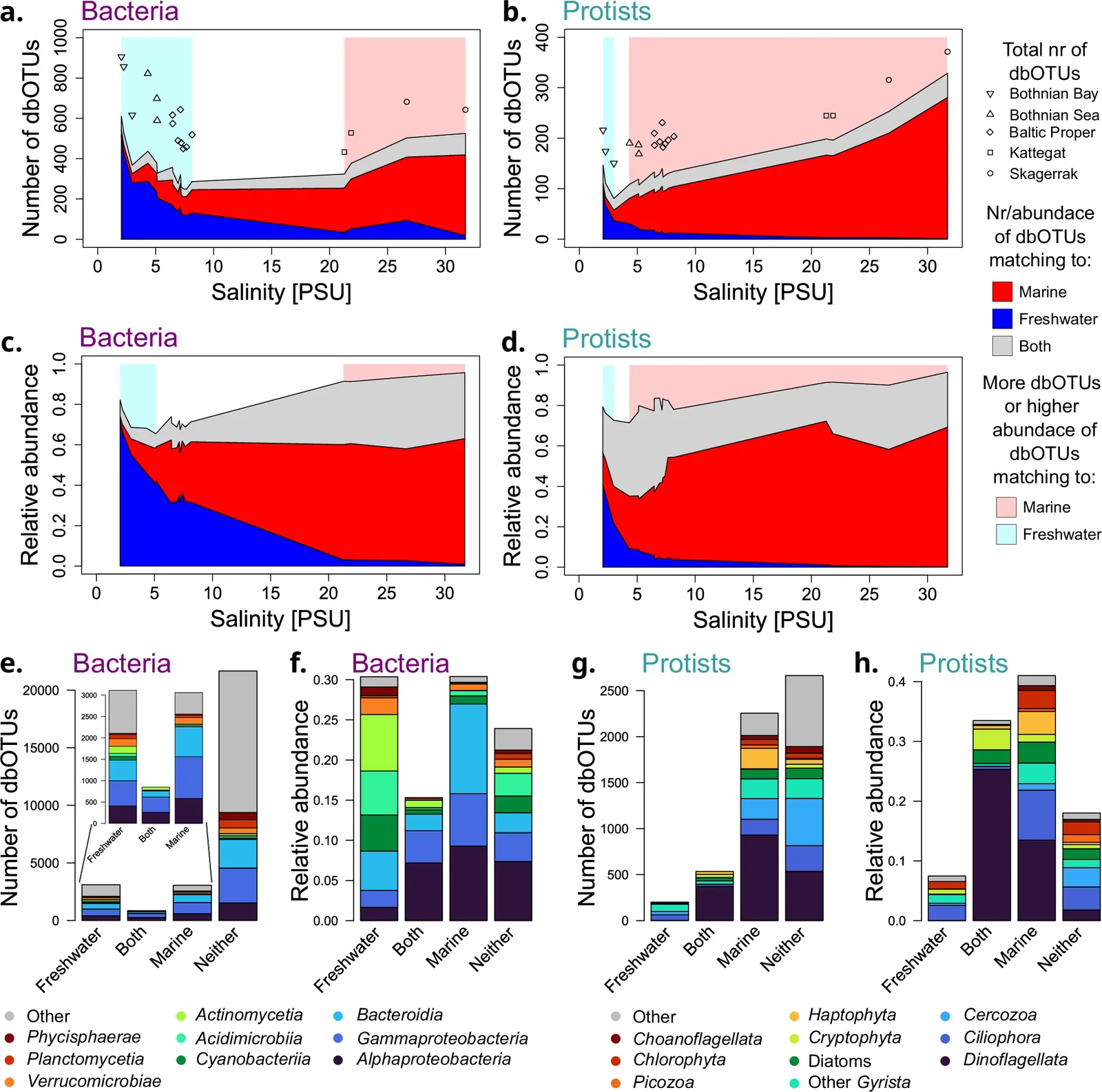
dbOTUs matching database marine and/or freshwater sequences. **a**, **b** The mean number of bacterial (**a**) and protist (**b**) dbOTUs matching marine or freshwater database sequences, or both. Mean total numbers of dbOTUs per station, based on the same rarefied count table, are labeled with sea-basin-coded shapes. The background color indicates salinity ranges at which all the stations had more dbOTUs matching freshwater or marine database sequences. **c**, **d** Relative abundance of bacterial (**c**) and protist (**d**) dbOTUs matching marine or freshwater database sequences, or both. Common legend for (**a**)–(**d**) is on the right-hand side of the panels. **e-f**. The number (**e**, **g**) and mean (across all samples) relative abundance (**f**, **h**) of bacterial (**e**, **f**) and protist (**g**, **h**) dbOTUs matching marine or freshwater database sequences, or both, or lacking a database match (“Neither”). The dbOTUs have been divided into bacterial classes and protist subdivisions. Only the classes/subdivisions which, on average, corresponded to >0.01 of reads across samples are shown. *Gyrista* has been divided into diatoms and other *Gyrista*. The legend for (**e-h**) are given in the bottom-most part of the figure. The marine and freshwater sequences were obtained from MiCoDa^68^ for bacteria and metaPR^2^^69^ for protists. Note: The sequences of ASVs comprising dbOTUs (259-537 bp for bacteria, 265-544 bp for protists) were compared to 90 bp database sequences. This was the length of available sequences in the 16S rRNA gene fragment database^68^, and we trimmed the 18S rRNA gene database sequences to the same length for comparability.

Across the salinity gradient, bacterial dbOTUs gradually transitioned from those matching freshwater database ASVs to those with marine database matches (Fig. 7a), consistent with the Herlemann study^14^. There were more freshwater- or marine-associated dbOTUs, respectively, on the lower and higher salinity sides of the Danish Straits. In contrast, for protists, while the number of marine-associated dbOTUs increases steadily with salinity, freshwater-associated dbOTUs outnumber them only in near-freshwater Bothnian Bay (Fig. 7b). These patterns held when the relative abundance, instead of the number of dbOTUs, was plotted (Fig. 7c, d).

Despite balancing the number and distribution of freshwater and marine reference samples (see Methods for details), we obtained more than three times as many marine (6867) as freshwater (1907) database protist ASVs. While a higher diversity of marine than freshwater protists has been reported, the true ratios are unlikely to be that high^70^. We thus downsampled the marine protist reference sequences to match the number of freshwater ASVs and repeated the analysis. However, the obtained pattern was congruent with the imbalanced analysis (Supplementary Fig. S11b, d). The number of freshwater and marine database ASVs for bacteria was very similar (12973 vs 11532), and downsampling to the same number of ASVs yielded near-identical results as the original analysis (Supplementary Fig. S11a, c).

The number and (mean across samples) relative abundance of bacterial freshwater- and marine-associated dbOTUs were nearly identical (Fig. 7e, f.), while marine-associated protists were much more numerous and abundant than freshwater-associated ones (Fig. 7g, h.). The proportion of freshwater- and marine-associated dbOTUs also differed between broad taxonomic groups of bacteria and protists. For example, only four dinoflagellate dbOTUs were freshwater-associated, compared to almost 1000 marine-associated ones (Fig. 7f–h). Consistently, most described *Dinoflagellata* species are marine^71,72^. The only protist subdivision with more freshwater- than marine-associated dbOTUs (12 vs 5) was *Cryptophyta*. Similarly, phylogenetic analyses have shown that cryptomonads more often transitioned from freshwater to marine environments than the other way round^73^. Meanwhile, freshwater- or marine-associated dbOTUs dominated different bacterial classes (Fig. 7e, f), in accordance with their spatial distribution within the Baltic Sea area (Fig. 1b, Supplementary Fig. S2a).

A substantial number of dbOTUs were associated with both freshwater and marine database ASVs, indicating overlap between these two biomes. We attribute this overlap to the short sequence length of the database fragments used (90 bp). For protists, where full 18S rRNA gene V4 fragment sequences were available in the database, we re-ran the analysis on the ASVs with lengths similar to those used in this study. While the pattern of freshwater-to-marine-associated dbOTUs remained the same, nearly none of the dbOTUs matched to both biomes (Supplementary Fig. S11e-f.). The freshwater-marine divide is even better documented for bacteria than for protists^1–7^. Thus, using longer 16S rRNA gene fragments would very likely also eliminate the matching to both freshwater and marine ASVs for bacteria.

For protists, the results presented here resemble the well-studied fish^30^ and macroalgal^31^ patterns^48^. Thus, they are highly suggestive of an underlying mechanism of local adaptation^31,41,48^. This would stand in stark contrast to the colonization of brackish-specialist bacteria after the Baltic Sea was formed^8^. However, the taxonomic resolution of metabarcoding hinders conclusions about local adaptation of protists. Follow-up studies using genomic analyses can test this hypothesis.

## Conclusions

We show that salinity has a stronger impact on bacterial than protist beta diversity, but that the reverse is true for alpha diversity. Alpha diversity (richness) of bacteria is maintained at similar levels across the brackish salinity gradient through a steady replacement of freshwater-associated lineages with marine-associated ones as salinity increases. Meanwhile, we show that marine-associated protists dominate in the Baltic Sea. As fewer and fewer of them can be found with decreasing salinity, alpha diversity drops. We also show that protist taxonomic units tend to have wider salinity niches, corroborating the beta diversity pattern.

These patterns are consistent with bacterial niches being dominated by brackish-water specialists that have dispersed from other brackish regions^8,12^, whereas the protists primarily represent locally adapted marine populations, analogous to the prevailing situation in Baltic Sea fish and macroalgae^31,41,48^. Colonization of the Baltic Sea by brackish specialist bacteria most likely required dispersal across hundreds or thousands of kilometers. Evidence from global oceans suggests that bacteria disperse more easily than protists across such long distances^74^. Similarly, multiple studies have shown more limited dispersal of aquatic protists than bacteria across shorter distances^75–77^. Given this, our findings that bacterial communities change more than protist ones across the Danish Straits (Fig. 2c, d), and that protist taxonomic units are more prone to occupy both sides of the straits (Fig. 6f, g), may appear counterintuitive. However, it is consistent with bacteria being less limited in long-range dispersal, allowing brackish specialists to colonize newly formed brackish basins and thus preventing local adaptation of populations from the neighboring waters from filling the niches. The idea of brackish-specialist bacteria colonizing newly formed brackish basins is supported by genomic analyses^8,12^. However, environment-coupled genomic data for brackish protists is too scarce^41^ to reach conclusions on the prevalence of local adaptation vs. long-distance recruitment. Thus, future environment-coupled genomic studies of diverse protists will challenge our hypothesis on differences in adaptation underlying the diversity patterns observed here.

Metabarcoding and metagenomics have shifted recent advances in understanding of aquatic microbial communities to focus much more on bacteria than on protists^78^. Our results strengthen the notion that conceptual frameworks developed for bacteria do not always translate into protists^78^. Salinity has been repeatedly described as the strongest factor affecting bacterial diversity, surpassed only by the separation of free-living and host-associated bacteria^1–4^. We show that this is unlikely to translate into protist diversity patterns, at the very least not within the brackish salinity spectrum. On the other hand, studies trying to assess whether bacteria or protist communities change more with the environment can overlook that the impact depends on the nature of the environmental change^16,26–28^. Here, we show that salinity has a stronger impact on bacterial than protist beta diversity, but the opposite is true for other factors. Ultimately, these patterns not only differ between bacteria and protists, but also within the groups. Therefore, we highlighted taxa, from families to subdivisions or classes, which most strongly contribute to the observed diversity patterns.

The spatiotemporal resolution of the study is a step forward a synthesis of knowledge from transect- and time-series-based diversity patterns. For example, we show that major bacterial and protist clades change not only in abundance but also in their seasonal dynamics across the Baltic Sea area. We also show that accounting for seasonal changes in bacterial alpha diversity is necessary to properly interpret the pattern across the salinity gradient. Alpha diversity increased in winter due to convective mixing, as observed before^17,36,38,66,67^. However, time-series-based studies consistently correlated the seasonal patterns of alpha diversity to day length^36,38,66,67^, and thus the importance of irradiation and other cryptic effects on the pattern is still being debated^38^. In our results, this correlation was poor, suggesting that vertical mixing dominates over the effects of irradiation on the common seasonal pattern of bacterial alpha diversity. Adding an additional temporal scale, we observed interannual spatiotemporal recurrence of community composition following the environmental gradients, consistent with previous results^28,53,63^. However, technical differences between the datasets confounded alpha diversity analysis. Thus, a standardized DNA-based scheme is needed to monitor rare microbial taxa, and our results can inform its development.

Using DNA metabarcoding across strong spatiotemporal gradients, we uncover patterns of microbial diversity and expand our understanding of previously observed ones. Ultimately, as Earth’s environments undergo rapid change, diverse microorganisms, often understudied groups, will be impacted and/or will impact the trajectory of brackish and other ecosystems. DNA-based monitoring covers the phylogenetic scope needed to understand those changes and respond to them. Understanding the ecoevolutionary processes behind the diversity patterns will be critical for the correct interpretation of their changes.

## Methods

### Data collection and sequencing

The 2019-2020 data come from Latz et al. 2024^49^. The collection and sequencing procedures are described in detail therein. In brief, samples were collected monthly or every second week, depending on the location, as part of the Swedish National Marine Monitoring Program. The samples for metabarcoding were collected using a depth-integrating hose, covering 0-10 m (except 0-5 m at RÅNEÅ-1, and 0-20 m at B1 and BY31). 500 ml of water from the hose sampling was filtered using 0.22 µm pore size. DNA was extracted using the Zymobiomics™ DNA miniprep kit following a modified protocol, and spike-in DNA was added^79^. The DNA was sequenced using Illumina™ MiSeq flow cells. For 16S rDNA gene-based metabarcoding of prokaryotes, primers targeting the hypervariable V3-V4 regions^14^, and for 18S metabarcoding of eukaryotes, primers targeting the V4 region^80^, were used. Phased primers^49,81^ were used for both 18S (on the forward primer) and 16S (on both primers) metabarcoding. Sequences of the primers and the details of the phasing strategy and sample indexing through a second PCR can be found in Latz et al. 2024^49^. Physico-chemical and geographic data have been obtained from the Swedish Meteorology and Hydrology Institute’s service SHARK: https://shark.smhi.se/en/ (some of the data used here are currently (at the time of publication) not available in SHARK, but can be found in the figshare repository for this study).

The 2015-2017 data were used for the first time for this study. The samples were stored at -80°C. DNA was extracted in the summer of 2017 and stored at -20°C until February 2023. The sampling and sequencing procedures were analogous to those for 2019-2020 data, with a few exceptions:

1. Volumes varied from 200 ml to 800 ml. According to our previous tests run in the same setting, volumes ≥ 200 ml should capture the microbial community structure equally well^49^.
2. DNA extraction was performed using Qiagen™ DNeasy PowerWater Kit, and not ZymoBIOMICS™ DNA Miniprep Kit with a small modification^79^ as for the 2019-2020 dataset.
3. No spike-in DNA was added.
4. Illumina™ NextSeq, rather than MiSeq system, was used.
5. For 18S, the initial use of 20 cycles (as in Latz et al.^49^) for the first PCR amplification was insufficient, leading to many library preparation failures. It was then re-run with 24 cycles, and the results of both runs were merged after processing the tables by summing up the counts in the output count table.
6. For 18S, reverse primers were also phased, following a sequence (CTACGA)CTTTCGTTCTTGATYRR, with versions of the primer starting from any base in the brackets included in the primer mix.
7. After library preparation, for most samples, 18S and 16S libraries were mixed and sequenced together. A cutadapt^82^-based pipeline was used to separate reads with only 16S or only 18S samples for downstream analysis, as described in the following section.

Samples from other projects, which we did not analyze in this study, were sequenced simultaneously and submitted to the European Nucleotide Archive (ENA) all together.

### Processing of sequencing data

The data processing for 2019-2020 samples has been described in detail in Latz et al. 2024^49^. In brief, the phased primer sequences were removed from the obtained reads using a modified version of a cutadapt^82^-based pipeline (original pipeline taken from: https://github.com/insect-biome-atlas/amplicon-multi-cutadapt). Only reads containing 16S or 18S primers were kept, which allowed us to separate reads from the two methods when the libraries were mixed. DADA2^83^ version 1.18.0 was used to denoise the data, infer amplicon sequence variants (ASVs), and taxonomically annotate them. For taxonomic annotation of 16S ASVs, we used 16S sequences from GTDB^84^ version R06-RS202-1 corrected for mislabeled sequences using SATIVA^85,86^. Annotation was additionally conducted using the SILVA database^87^ version 138.1 to identify plastids and mitochondria. The 18S ASVs were annotated using the PR2 database^88^ version 5.0.0.

The 2015-2017 data was analyzed the same way, with the only difference being that the denoising and ASV inference with DADA2 was performed using the nf-core/ampliseq pipeline^89^ version 2.7.0 instead of an in-house R script. We kept the DADA2 parameters as in Latz et al. 2024^49^.

We further combined the 2015-2017 and 2019-2020 data. We matched ASVs using exact matching. On the merged ASV-sequence list and count table, we ran an additional chimera removal step using UCHIME v1^90^ and clustered the remaining ASVs based on their simultaneous genetic and distribution similarity using dbOTU3^50^. Both these steps were performed using an in-house pipeline available at https://github.com/kjurdzinski/chimera_dbOTU_pipeline. Default parameters were used for UCHIME, and for dbOTU3, only the maximum genetic distance for 16S has been changed to 3% (instead of the default 10%). dbOTU aims to identify multiple ASVs stemming from the same population, i.e., multiple rRNA gene copies with some nucleotide differences encoded in the same genome, and keep only one of them. This procedure is particularly important for alpha diversity analyses, in which multiple distinct rRNA gene copies could inflate the observed richness. We called the resulting sequence clusters dbOTUs (distance-based operational taxonomic units, as inferred using dbOTU3^50^). This clustered the 122,371 and 49,600 ASVs for bacteria and protists into 48,961 and 9,909 dbOTUs,

For both 16S and 18S, we removed dbOTUs with no annotation at the phylum/supergroup taxonomic level. From 16S dbOTUs, *Archaea*, plastids, and mitochondria were removed. Thus, the remaining dbOTUs should correspond to bacteria. From 18S dbOTUs, animals (*Metazoa*), *Fungi*, land plants (*Embryophyceae*), and selected macroalgae (*Phaeophyceae* and Rhodophyta excluding *Rhodella* and *Cyanidiales*) were removed. Thus, the remaining dbOTUs were primarily protists.

We kept spike-in dbOTUs in a separate table and used them only to obtain spike-in corrected abundance, i.e., the inferred number of rDNA copies per mililiter. The rDNA copies per liter were calculated according to the equation:

$$	{rDNA}\,{copies}\,{per}\,{milliliter} =\frac{{dbOTU}\,{counts}\,}{{spike}-{in}\,{counts}} \\ 	 \times \frac{{amount\;of\;spike}-{in}\,{added}\;[g]}{{volume}\,{water}\,{sampled}\;[{ml}]\times {spike-in}\,{molar}\;{mass}\;[g/{mol}]}\times 6.022\times {10}^{23}$$

We have excluded over- and under-sequenced samples to remove the amplification errors (including distorted relative abundances) and not to rarefy to a too low read number during rarefaction. We have excluded three samples with fewer than 30,000 reads for bacteria, as well as one sample with more than 2,000,000 reads. Moreover, we have excluded two samples from 2019-2020 with more than 200,000 protist reads (this number of reads was normal for 2015-2017 data since Illumina™ NextSeq was used instead of MiSeq, yielding overall higher numbers of reads). We have chosen the thresholds based on visual examination of the distribution of the sequencing depths, removing outliers. Additionally, one sample (20191015_263953_1) with suspected contamination has been excluded.

For comparability of results for protists and bacteria, we used only the samples with data available from both 16S and 18S amplicon sequencing (excluding the under- and over-sequenced samples) for all further analyses. Additionally, in two samples (station RÅNEÅ-2 on 2019-01-29 (sample ID: 20190129_263856_1) and B7 on 2019-09-17 (sample ID: 20190917_264450_1)) 18S spike-in addition failed. Those samples were excluded from analyses in which spike-in normalization was used, except for bacteria-only focused analyses in Fig. 1b.

### Abundance, alpha and beta diversity spatiotemporal patterns

For the spatiotemporal analysis of abundance, beta- and alpha diversity across the samples from 2019-2020, we removed the samples from stations H4, BY29 / LL 19, and SR3. Those stations were sampled at only a few unevenly distributed occasions, and their exclusion/inclusion at certain seasons could obscure seasonal patterns.

All the statistical analyses and data visualization were performed using R version 4.2.2^91^. Inkscape^92^ was used to assemble and edit multi-panel figures.

The multivariate analyses were performed using vegan R package^93^, and all the functions in this paragraph can be found there unless another package is explicitly stated. Bray–Curtis distances between communities were obtained using rarefaction via the avgdist function. Those distances were used for Principal Coordinate Analysis (PCoA) and distance-based Redundancy Analysis (dbRDA), performed with, respectively, capscale (without setting constraints) and dbrda functions. We chose dbRDA to base the constrained analysis on Bray-Curtis distances, which are a more robust metric of ecological dissimilarity than Euclidean distances used in redundancy analysis (RDA) or chi-square distances used in Canonical Correspondence Analysis (CCA)^94^. Variance partitioning was performed using rdacca.hp R package^95^. Partial dbRDA was performed by using the special term Condition() in the dbRDA function’s formula. Families were positioned on the ordination plots by calculating weighted averages (sppscores function). Only the families deviating (Bonferroni-corrected *P*-value < 0.05) from normal distribution around the center of the dbRDA coordinate system were shown on the plots. To find the corresponding two-dimensional ellipsoid confidence intervals, aspace^96^ package was used. Whenever only samples were visualized, their ordination scores were scaled using the eigenvalues, and symmetric scaling was used if both the families and the samples were visualized (using, respectively, “sites” and “symmetric” scaling options of the plotting functions). For dbRDA and variance partitioning, we used a subset of 228 (out of 246) samples with all the environmental variables of interest available. For more informative visualization, to keep the sample structuring visible despite very high family scores, we divided the family scores in half. We chose day length as one of the factors, as it has been connected with seasonal changes in bacterial alpha diversity^36,38^ and, together with temperature, should catch the full scope of seasonality (as temperature increase is lagging behind day length).

dbOTU richness was calculated as the mean number of dbOTUs detected in a sample over 100 rarefaction iterations. Variance partitioning was performed using rdacca.hp R package^95^.

For both beta and alpha diversity estimation, in each rarefaction iteration, we rarefied the bacterial 16S reads to 34493 per sample, and the protists 18S reads to 8009 per sample.

For deepwater taxa detection, we used the representative sequences of 99% average nucleotide identity OTUs (operational taxonomic units) detected in samples from the summer of 2008 and coming from a previous study^17^. The 2008 summer transect included sampling across different depths, and the deepwater OTUs in this study were present exclusively at depths ranging from 11 to 300 m, classified as mesopelagic in the original study^17^. We connected the OTUs from the original, transect-based study to ASVs from the monitoring data used here, using the 99% nucleotide identity threshold in VSEARCH^97^. Despite substantial methodological differences, we found a match for 36.6% of the OTUs and 16.1% of dbOTUs at a 99% nucleotide identity threshold. We deemed an OTU and a dbOTU to be matching if their representative sequences (most abundant ASV for a dbOTU) matched in the VSEARCH output. For comparing the number of OTUs from deepwater, surface water, and both (Fig. 5a) in winter and summer, we used the chi-squared test as implemented in the prop.test function in R^98^. We excluded Bothnian Bay from the analyses since there were no deepwater samples from this region in the transect-based dataset.

### Niche breadths

We have estimated the niche breadths with the nichePOSNB function of the ecospat package^99^. This function calculates niche breadth as the standard deviation of a parameter’s values weighted by the relative abundance of a taxon.

We used the estimated niche breadths to build a Bayesian model in JAGS^100^, using the R package rjags^101^. The parameters, probabilities, and predictions were calculated based on every fifth out of 10000 MCMC iterations. We modeled salinity niche breadth based on the normal/Gaussian distribution:

$${salinity}{\_}{niche}{\_}{breadt}{h}_{i}\sim {Normal}({\mu }_{i},\,\sigma )$$

where ${salinity\_niche\_breadt}{h}_{i}$ is the estimated salinity niche breadth for a dbOTU, σ is the standard deviation of the distribution from which these values are modeled to be sampled, while ${\mu }_{i}$ is the mean of the distribution. ${\mu }_{i}$ was calculated based on the equation:

$$	{\mu }_{i}=(1-{if}{\_}{protis}{t}_{i})\times ({\alpha }_{{bacterium}}+{\beta }_{{temperature}{\_}{bacterium}}\times {temperature}{\_}{niche}{\_}{breadt}{h}_{i}+ \\ 	 {\beta }_{{DIN}{\_}{bacterium}}\times {DIN}{\_}{niche}{\_}{breadt}{h}_{i}\,+\,{\beta }_{{phosphate}{\_}{bacterium}} \\ 	 \times {phosphate}{\_}{niche}{\_}{breadt}{h}_{i}\,{\beta }_{{day}{\_}{length}{\_}{bacterium}}\times {day}{\_}{length}{\_}{niche}{\_}{breadt}{h}_{i})+{if}{\_}{protis}{t}_{i} \\ 	 \times ({\alpha }_{{protist}}+\,{\beta }_{{temperature}{\_}{protist}}\times {temperature}{\_}{niche}{\_}{breadt}{h}_{i}\,+{\beta }_{{DIN}{\_}{protist}} \\ 	 \times {DIN}{\_}{niche}{\_}{breadt}{h}_{i}+{\beta }_{{day}{\_}{length}{\_}{protist}}\times {day}{\_}{length}{\_}{niche}{\_}{breadt}{h}_{i})$$

The prior for each parameter was set to 0, corresponding to a lack of knowledge of any effect on the dependent variable.

### Proportion salinity barrier crossing dbOTUs

We analyzed which dbOTUs were found in at least one sample from near-marine stations (Å17, SLÄGGÖ) and from comparable stations in the Baltic Sea (B1 and BY31 LANDSORTSDJ). These samples were far away from the Danish Straits, imposing the salinity barrier, reducing the risk of false detection of dbOTUs whose DNA or dead/dormant cells were carried with the water flow or aerosols across the straits. The two stations on each side of the barrier comprised one near-coast and one offshore station. For each station but the least sampled (Å17 and B1), we chose a subset of samples (n = 22), keeping samples closest in time to the ones collected at the least sampled stations. Whenever available, those samples should correspond to the same monitoring cruises.

To reduce detection biases, 16S and 18S rRNA gene reads were downsampled to the same level (10,863) across the samples.

The Bayesian models were built and implemented in JAGS^100^, using the R package rjags^101^. The parameters, probabilities, and predictions were calculated based on every fifth out of 10000 MCMC iterations.

The model for the probability of crossing the salinity barrier was optimized based on the Bernoulli distribution:

$${if}{\_}{crossed}{\_}{{barrier}}_{i} \sim {Bernoulli}\left({p}_{i}\right)$$

where for each dbOTU *i*, ${if\_crossed\_barrie}{r}_{i}$ is a binary variable = 1 if it is a barrier-crossing dbOTU. ${p}_{i}$ was calculated according to the formula

$${logit}({p}_{i})= 	 \, (1-{if}{{\_}}{protis}{t}_{i})\\ 	 \times ({\alpha }_{{bacterium}}+\,{\beta }_{{occurence}{{\_}}{bacterium}} \times \max {{\_}}{occurence}{{\_}}{one}{{\_}}{sid}{e}_{i})\\ 	 +{if}{{\_}}{protis}{t}_{i}\times ({\alpha }_{{protist}} +{\beta }_{{occurence}{{\_}}{protist}}\times \max {{\_}}{occurence}{{\_}}{one}{{\_}}{sid}{e}_{i})\,$$

where ${if\_protis}{t}_{i}$ is a binary variable = 1 if the dbOTU *i* is a protist. $\max {\_occurence\_one\_sid}{e}_{i}$ is the maximum out of the numbers of samples in which the dbOTU *i* was found at one side of the salinity barrier (in salinity <9 PSU or >15 PSU). The other variables are the parameters of the model.

The model for total occupancy was built based on the Poisson distribution

$${total}{\_}{occupanc}{y}_{i}\,\sim \,{Poisson}({\lambda }_{i})$$

where ${total\_occupanc}{y}_{i}$ is the total number of samples in which dbOTU *i* was found. ${\lambda }_{i}$ was calculated according to the formula:

$${\lambda }_{i}= 	 \, \max {\_}{occurence}{\_}{one}{\_}{sid}{e}_{i}\,+\,(1-{if}{\_}{protis}{t}_{i})\\ 	 \times \left({\alpha }_{{bacterium}} +\,{\beta }_{{bacterium}}\times \log (\max {\_}{occurence}{\_}{one}{\_}{sid}{e}_{i})\right.\\ 	 +{if}{\_}{protis}{t}_{i}\times ({\alpha }_{{protist}} +\,{\beta }_{{protist}}\times \max {\_}{occurence}{\_}{one}{\_}{sid}{e}_{i})$$

with ${if\_protis}{t}_{i}$ and $\max {\_occurence\_one\_sid}{e}_{i}$ having the same meaning as described above. The occupancy at the less occupied side of the salinity barrier was calculated by subtracting $\max {\_occurence\_one\_side}$ from the predicted λ.

The prior for each parameter was set to 0, corresponding to a lack of knowledge of any effect on the dependent variable.

### Matching to freshwater and marine ASVs

For bacteria, we downloaded 16S rRNA gene V4 fragment sequences from MiCoDa v2 falling into “saltwater” and “lake” categories (“Environment - broad scale”). We then used entrez_search() and entrez_summary() functions from the rentrez R package to obtain metadata for the samples. We selected samples from less than 10 m depth, and more than 34 PSU salinity for saltwater samples, and “Freshwater” as “envrionment_feature” for the lake samples. We thus identified 182 marine and 918 freshwater samples, and downsampled the freshwater samples to match the number of marine samples. Altogether, we got 11532 marine ASVs and 12973 freshwater ASVs, with 167 ASVs found both in freshwater and marine samples.

For protists, we downloaded 18S ASVs from the metaPR^2^ database version 3^69^ on October 20th 2025. We have chosen the “oceanic” (hereafter referred to as marine) and “freshwater” ecosystems (“freshwater lake” and “freshwater river”), all size fractions, “surface” depth level, and only ASVs with a minimum of 100 reads in the database (default option). Since all the downloaded freshwater samples came from the temperate climate, we limited the marine samples to the temperate climate as well. Furthermore, we limited the marine samples to the ones with recorded salinity above 34 PSU. We were then left with 238 marine and 208 freshwater samples. To equalize the number of samples per biome, we randomly chose 208 out of the 238 marine samples. Out of the downloaded ASVs, we chose only the protist ones, as described in the “Processing of sequencing data” section. We then rarefied the count tables to the minimum sampling depth (1161 reads) and kept only the ASVs with reads remaining after rarefying. We also removed the one ASV found in both freshwater and marine samples from the downstream analyses. Overall, we obtained 5568 marine and 1913 freshwater ASVs. We kept both all the marine ASVs and a list with as many marine ASVs as available for the freshwater biome, chosen randomly, with the probability of an ASV being chosen weighted by its average abundance in the database sample. We then trimmed the ASVs to 90 bp length to be comparable with matching to MiCoDa for bacteria.

We have connected the ASVs from our study to the database ASVs, obtaining perfect matches (100% nucleotide identity threshold) with VSEARCH^97^. We considered a dbOTU to be marine or freshwater if the representative ASV had a perfect match in the reference database.

### Reporting summary

Further information on research design is available in the Nature Portfolio Reporting Summary linked to this article.

## Supplementary information

Transparent Peer Review file

Supplementary Figs.

Reporting Summary

## Data Availability

The 2019-2020 data has been published before as a data paper^49^, and is available in the European Nucleotide Archive under the study accession code PRJEB55296. The 2015-2017 raw sequencing data is available in the European Nucleotide Archive under the study accession code https://identifiers.org/ena.embl:PRJEB84926. The ASVs identified in the 2015-2017 sequencing data, together with a count table and contextual data, can be accessed through the ASV portal at the Swedish Biodiversity Data Infrastructure (SBDI). Subsequently, the ASVs, count tables, and contextual information are available at the Global Biodiversity Information Facility (GBIF), under accessions https://www.gbif.org/dataset/fbf49d0a-df4c-4b89-ba71-143bf6430a91 for 16S rRNA gene and https://www.gbif.org/dataset/9a228813-6288-4aba-a8f8-480e425b5f62 for 18S rRNA gene.

## Code availability

The code used to analyze the data and generate the figures, together with intermediate processing output, can be found at https://doi.org/10.17044/scilifelab.28673273.
