# Supplementary material for: DNA-based monitoring uncovers contrasting salinity-driven diversity patterns of bacterial and protist plankton in the Baltic Sea area
Source: Commun Biol. 2026 Oct 7;9:1308. doi: 10.1038/s42003-026-10977-4 (PMC13646260; doi:10.1038/s42003-026-10977-4)
Supplement: Supplementary file 1 [file 42003_2026_10977_MOESM1_ESM.pdf]

nature portfolio

Peer Review File

DNA-based monitoring uncovers contrasting salinity-driven diversity patterns of bacterial and protist plankton in the Baltic Sea area

Corresponding Author: Professor Anders Andersson

This manuscript has been previously reviewed at another journal. This document only contains information relating to versions considered at Communications Biology.

This file contains all reviewer reports in order by version, followed by all author rebuttals in order by version.

Version 0:

Reviewer comments:

Reviewer #1

(Remarks to the Author)
General Comments

The authors surveyed bacterial and protistan communities along salinity gradients in the Baltic Sea over two sampling periods (2015–2017 and 2019–2020). The study found that community composition in both groups is strongly associated with salinity, although the proportion of variation explained by salinity is substantially greater for bacteria than for protists. The analyses further suggest that bacterial and protistan taxa exhibit distinct salinity niche distributions. Specifically, bacterial communities in this region appear to be largely composed of distinct freshwater and marine lineages, whereas many protistan taxa consist of larger marine species with broader salinity tolerances.
I find this overall storyline interesting and potentially important, as it provides a plausible explanation for the contrasting responses of bacterial and protistan communities to salinity gradients. In my opinion, the dataset is valuable and the central observations have the potential to make a meaningful contribution to our understanding of microbial community assembly along salinity gradients.

However, the manuscript in its current form requires substantial revision. As written, the manuscript appears largely descriptive. Large portions of the Results and Discussion are devoted to methodological and statistical considerations, often at the expense of biological interpretation. In several places, the manuscript discusses analytical approaches, diversity metrics, and technical limitations in considerable detail without clearly linking them to the ecological questions being addressed. This makes the manuscript difficult to follow and weakens the overall narrative. I recommend substantially reducing methodological discussion outside the Methods section and refocusing the Results and Discussion on the ecological implications of the findings.

In addition, the authors refer to this work as illuminating "eco-evolutionary processes" in both the manuscript title and response letter. However, I found limited discussion or analysis directly addressing evolutionary mechanisms. The study primarily focuses on ecological patterns of community composition along environmental gradients. If the authors intend to retain the eco-evolutionary framing, additional justification and discussion are needed to explain how the presented results support that interpretation. Alternatively, a more ecology-focused title may better reflect the scope of the analyses. For example, a title such as "Contrasting salinity niche distributions of bacterial and protist plankton along a Baltic Sea gradient" may more accurately capture the main findings of the study.

Finally, the figures would benefit from substantial revision. Several figures appear to have insufficient resolution, making text and graphical elements difficult to read. Font sizes are inconsistent among subpanels, and some labels are disproportionately large relative to the figure dimensions. In several cases, figure legends and annotations overlap with the plotted data, further reducing readability.

More generally, many of the figures appear overly complex and visually crowded, making it difficult for readers to identify the

main patterns. Although salinity is clearly a central variable in this study, I do not find it helpful to scale symbol sizes according to salinity throughout multiple figures. This additional visual layer often obscures rather than clarifies the underlying results and contributes to an unnecessarily busy presentation. In many cases, simpler figure designs would communicate the key findings more effectively. I therefore recommend careful revision of all figures.

Specific Comments
(Because the track-changes version is difficult to read, all line references below refer to the clean manuscript version without tracked changes).

Lines 114–116: The rationale for using dbOTUs instead of ASVs is unclear. The Methods section (Lines 568–569) does not explain why dbOTUs were preferred or how this choice benefits the current study. Please provide a justification.

Lines 120–123: The spike-in approach is already described in the Methods section. Reintroducing methodological details here is unnecessary and disrupts the flow of the Results/Discussion.

Lines 124–127: This two-sentence paragraph is unusually short and could likely be merged with the surrounding text.

Lines 165–166: I disagree with the statement regarding the utility of relative abundance data. Relative abundance is inherently compositional and constrained by a zero-sum structure, which can obscure ecological patterns rather than reveal them. Therefore, I do not think relative abundance data are particularly well-suited for detecting succession or species competition.

Lines 169–172: Figure S3 is difficult to interpret. With five stations sampled monthly over twelve months, a Bray–Curtis distance analysis should generate approximately 1,770 pairwise comparisons. However, the figure contains far fewer points. Please clarify how the distances were calculated and why only a subset of comparisons is shown.

In addition, the scatterplots show two distinct clusters, but these are not discussed in the text. The background color gradient is also not explained in the figure legend.

Lines 173–174: The description of the ordination results does not adequately capture the dominant patterns.

For the bacterial community, PCoA axis 1 primarily separates the Skagerrak and Kattegat stations (salinity consistently >20 PSU) from the remaining stations (<10 PSU), indicating a strong geographic and salinity-related gradient. PCoA axis 2 appears to capture temporal variation.
Thus, spatial/geographic variation appears more important than seasonal variation for bacterial communities. Protistan communities show a similar trend, although the geographic separation is less pronounced.

Lines 175–178: Why was dbRDA selected instead of conventional RDA or CCA? The manuscript does not provide a justification for this choice. Furthermore, it is unclear whether environmental variables were standardized prior to analysis. If standardization was not performed, the conclusion that salinity is the most important environmental variable may simply reflect its high variability relative to other variables.

Lines 180–183: If seasonal variation is a primary focus, why was day length selected as the seasonal indicator instead of directly incorporating sampling month or season? This choice should be justified in the Methods section rather than discussed only in the Results.

Lines 197–198: This sentence is unclear and should be rewritten for clarity.

Lines 227–228: This statement appears highly speculative and is not directly supported by the presented data. I recommend removing it or providing stronger evidence.

Lines 229–248: These two paragraphs focus primarily on technical and statistical issues associated with alpha-diversity comparisons between samples collected during 2016–2017 and 2019–2020. There is little biological interpretation. Unless a stronger ecological explanation can be developed, I suggest substantially shortening or moving this section to the supplimantary.

Lines 577–582: This paragraph is confusing because dbOTUs and ASVs appear to be used interchangeably, despite representing different taxonomic units. The terminology should be used consistently throughout the manuscript.

Figure 1b and 1c: Bacterial abundance is typically reported as cells mL^{-1} rather than cells L^{-1}. Consider changing the unit for easier comparison with previous studies. The abundance estimates suggest that bacterial and protistan abundances are of similar magnitude. This is unexpected in most natural aquatic systems, where bacterial abundances are typically orders of magnitude higher than those of protists. Please provide an explanation for this pattern.

Figure 1d and Figure 3b: These figures are not adequately described in the text. In addition, scaling symbol size by salinity makes the figures unnecessarily busy and difficult to interpret. Since pairwise correlations of environmental variables are already provided in the Supplementary Information, simple boxplots may communicate the results more effectively.

Figure 4a and 4b: I do not think it is necessary to scale symbol size according to salinity, given that salinity is already represented on the x-axis.

Reviewer #2

(Remarks to the Author)
I had a look at the authors' response to reviewer #1 specifically and I feel that the authors addressed this reviewer's concerns appropriately. I also feel that many of the concerns the authors raised around salinity are due to them not accounting for the fact that this study was conducted in brackish water.

Version 1:

Reviewer comments:

Reviewer #1

(Remarks to the Author)
The authors have adequately addressed all of my comments and concerns. I have no further questions.

Open Access This Peer Review File is licensed under a Creative Commons Attribution 4.0 International License, which permits use, sharing, adaptation, distribution and reproduction in any medium or format, as long as you give appropriate credit to the original author(s) and the source, provide a link to the Creative Commons license, and indicate if changes were made.
In cases where reviewers are anonymous, credit should be given to 'Anonymous Referee' and the source.
The images or other third party material in this Peer Review File are included in the article's Creative Commons license, unless indicated otherwise in a credit line to the material. If material is not included in the article's Creative Commons license and your intended use is not permitted by statutory regulation or exceeds the permitted use, you will need to obtain permission directly from the copyright holder.
To view a copy of this license, visit https://creativecommons.org/licenses/by/4.0/

Reviewer #1:

General Comments

The authors surveyed bacterial and protistan communities along salinity gradients in the Baltic Sea over two sampling periods (2015–2017 and 2019–2020). The study found that community composition in both groups is strongly associated with salinity, although the proportion of variation explained by salinity is substantially greater for bacteria than for protists. The analyses further suggest that bacterial and protistan taxa exhibit distinct salinity niche distributions. Specifically, bacterial communities in this region appear to be largely composed of distinct freshwater and marine lineages, whereas many protistan taxa consist of larger marine species with broader salinity tolerances.
I find this overall storyline interesting and potentially important, as it provides a plausible explanation for the contrasting responses of bacterial and protistan communities to salinity gradients. In my opinion, the dataset is valuable and the central observations have the potential to make a meaningful contribution to our understanding of microbial community assembly along salinity gradients.

Reply: We appreciate the reviewer's comments on the appeal and importance of our work.

However, the manuscript in its current form requires substantial revision. As written, the manuscript appears largely descriptive. Large portions of the Results and Discussion are devoted to methodological and statistical considerations, often at the expense of biological interpretation. In several places, the manuscript discusses analytical approaches, diversity metrics, and technical limitations in considerable detail without clearly linking them to the ecological questions being addressed. This makes the manuscript difficult to follow and weakens the overall narrative. I recommend substantially reducing methodological discussion outside the Methods section and refocusing the Results and Discussion on the ecological implications of the findings.

Reply: We have now reduced the methodological discussion in accordance with the specific comments of the reviewer.

In addition, the authors refer to this work as illuminating "eco-evolutionary processes" in both the manuscript title and response letter. However, I found limited discussion or analysis directly addressing evolutionary mechanisms. The study primarily focuses on ecological patterns of community composition along environmental gradients. If the authors intend to retain the eco-evolutionary framing, additional justification and discussion are needed to explain how the presented results support that interpretation. Alternatively, a more ecology-focused title may better reflect the scope of the analyses. For example, a title such as "Contrasting salinity niche distributions of bacterial and protist plankton along a Baltic Sea gradient" may more accurately capture the main findings of the study.

Reply: We suggest “DNA-based monitoring uncovers contrasting salinity-driven diversity patterns of bacterial and protist plankton in the Baltic Sea area” as a new title that encompasses the focus and the scope of this study.

Finally, the figures would benefit from substantial revision. Several figures appear to have insufficient resolution, making text and graphical elements difficult to read. Font sizes are inconsistent among subpanels, and some labels are disproportionately large relative to the figure dimensions. In several cases, figure legends and annotations overlap with the plotted data, further reducing readability.

Reply: We have now submitted the figures as PDFs for higher resolution to be accessible to the reviewer, and revised them according to the reviewer’s specific comments. We also made sure that all the supplementary figures are in vector image quality, since we do not provide separate PDFs for them.

Even if the legends overlap with the plotting area, they do not overlap with the data. We have now made sure to minimize the overlap of labels with the data and that they do not make any data points undetectable. Additionally, we have made the following changes to the figures following the reviewer’s general comments:

Fig. 1 - changed x-axis labels to the same size in panel d.
Fig. 2 - moved labels in panels c-f to decrease the overlap with the data.
Fig. 3 - decreased the label sizes in the PCoA plots (now panels b-c). Also moved parts to the supplementary.
Fig. 4 - reduced the size of y-axis labels in all panels and x-axis labels in panels a-b. Also moved parts to the supplementary.
Fig. 5 - reduced the size of all axis labels.
Fig. 6 - no changes.
Fig. 7 - reduced the size of the legend for panels a-d.

More generally, many of the figures appear overly complex and visually crowded, making it difficult for readers to identify the main patterns. Although salinity is clearly a central variable in this study, I do not find it helpful to scale symbol sizes according to salinity throughout multiple Dfigures. This additional visual layer often obscures rather than clarifies the underlying results and contributes to an unnecessarily busy presentation. In many cases, simpler figure designs would communicate the key findings more effectively. I therefore recommend careful revision of all figures.

Reply: Repeated use of ordination methods makes putting salinity on one of the axes infeasible for most of the figures. We reserved color for seasonality, as it is possible to make a circular color scale but not a circular size scale. Thus, to represent a continuous value, we were left with size for salinity. To complement this continuous scale, we have marked the sea basins with different shapes, and as the basins represent different salinity values, with only the Kattegat and Skaggerak ranges overlapping, the shapes provide an additional visual clue on the salinity

gradient. We understand that a size scale is not the easiest to read, but we stand behind our choice.

As for the complexity of the figures, we agree that it would make the figures easier to read. There is, however, a tradeoff between readability and providing enough context for the reader to critically assess the connection between the claims we make and the results. Nevertheless, we moved parts of Fig. 3 and Fig. 4 to the supplementary material to make them less busy.

Specific Comments
(Because the track-changes version is difficult to read, all line references below refer to the clean manuscript version without tracked changes).

Reply: In accordance with the reviewer's previous choice, we provide line number from the version without tracked changes.

Lines 114–116: The rationale for using dbOTUs instead of ASVs is unclear. The Methods section (Lines 568–569) does not explain why dbOTUs were preferred or how this choice benefits the current study. Please provide a justification.

Reply: We now justify it as follows:

"*dbOTU aims to identify multiple ASVs stemming from the same population, i.e., multiple rRNA gene copies with some nucleotide differences encoded in the same genome, and keep only one of them. This procedure is particularly important for alpha diversity analyses, in which multiple distinct rRNA gene copies could inflate the observed richness.*" (lines 574-678)

Lines 120–123: The spike-in approach is already described in the Methods section. Reintroducing methodological details here is unnecessary and disrupts the flow of the Results/Discussion.

Reply: We believe that this information helps the reader to understand the following paragraphs. Nevertheless, we rephrased it to make the relevance clearer:

"*Because the samples also contained a known amount of synthetic spike-in DNA amplifiable by the metabarcoding primers, absolute abundances of individual dbOTUs could be estimated.*"

Lines 124–127: This two-sentence paragraph is unusually short and could likely be merged with the surrounding text.

Reply: We appreciate the suggestion, but we believe that having a general paragraph followed by one about phytoplankton and one about heterotrophic bacteria serves the readability of the paper, even if the first paragraph is short.

Regardless, we now expanded the paragraph in response to another comment (lines 126-132).

Lines 165–166: I disagree with the statement regarding the utility of relative abundance data. Relative abundance is inherently compositional and constrained by a zero-sum structure, which can obscure ecological patterns rather than reveal them. Therefore, I do not think relative abundance data are particularly well-suited for detecting succession or species competition.

Reply: We agree that many ecological patterns can be obscured by the nature of relative abundance. However, by looking at how the abundance of different taxa changes relative to others, one can assess, e.g., the replacement of taxa in a successive manner. Also, by looking at changes in relative abundance and having some knowledge about resources used by present taxa, one can identify when one taxon outcompetes another, regardless of the total resources available.

Nevertheless, we found this sentence redundant and removed it.

Lines 169–172: Figure S3 is difficult to interpret. With five stations sampled monthly over twelve months, a Bray–Curtis distance analysis should generate approximately 1,770 pairwise comparisons. However, the figure contains far fewer points. Please clarify how the distances were calculated and why only a subset of comparisons is shown.

In addition, the scatterplots show two distinct clusters, but these are not discussed in the text. The background color gradient is also not explained in the figure legend.

Reply: This is a density map; the most outlying sample-sample comparisons were shown as single points, and the rest have been color-coded based on the density of points with the corresponding values. We have now removed the single points and added a legend to the density plot. We also specified in the figure description that it is a density plot.

This is based on 246 samples, thus (246*245)/2=30,135 comparisons.

Lines 173–174: The description of the ordination results does not adequately capture the dominant patterns.

For the bacterial community, PCoA axis 1 primarily separates the Skagerrak and Kattegat stations (salinity consistently >20 PSU) from the remaining stations (<10 PSU), indicating a strong geographic and salinity-related gradient. PCoA axis 2 appears to capture temporal variation.
Thus, spatial/geographic variation appears more important than seasonal variation for bacterial communities. Protistan communities show a similar trend, although the geographic separation is less pronounced.

Reply: Thank you for pointing this out. We modified the sentence; it now reads:

"*Principal Coordinate Analysis (PCoA) showed stronger geographic than seasonal structuring of bacterial communities, while for protists the effects of geography and season were comparable*" (lines 177-179)

Lines 175–178: Why was dbRDA selected instead of conventional RDA or CCA? The manuscript does not provide a justification for this choice.

Note: We divided this comment from the reviewer into two for clarity of the response.
Reply: We now added an explanation in the methods section:

"*We chose dbRDA to base the constrained analysis on Bray-Curtis distances, which are a more robust metric of ecological dissimilarity than Euclidian distances used in redundancy analysis (RDA) or chi-square distances used in Canonical Correspondence Analysis (CCA) (Faith et al. 1987)*" (lines 627-631)

Furthermore, it is unclear whether environmental variables were standardized prior to analysis. If standardization was not performed, the conclusion that salinity is the most important environmental variable may simply reflect its high variability relative to other variables.

Reply: dbRDA is based on correlations between the distances and environmental variables, and correlations between environmental variables. Thus, a linear transformation, such as scaling by variance, will not change the results, even if the variables are divided by different values (e.g., standard deviations). Consistently, when we run rdacca.hp after standardization (i.e., on z-scores of the environmental variables, instead of raw values), we get the same results as before, as shown below:

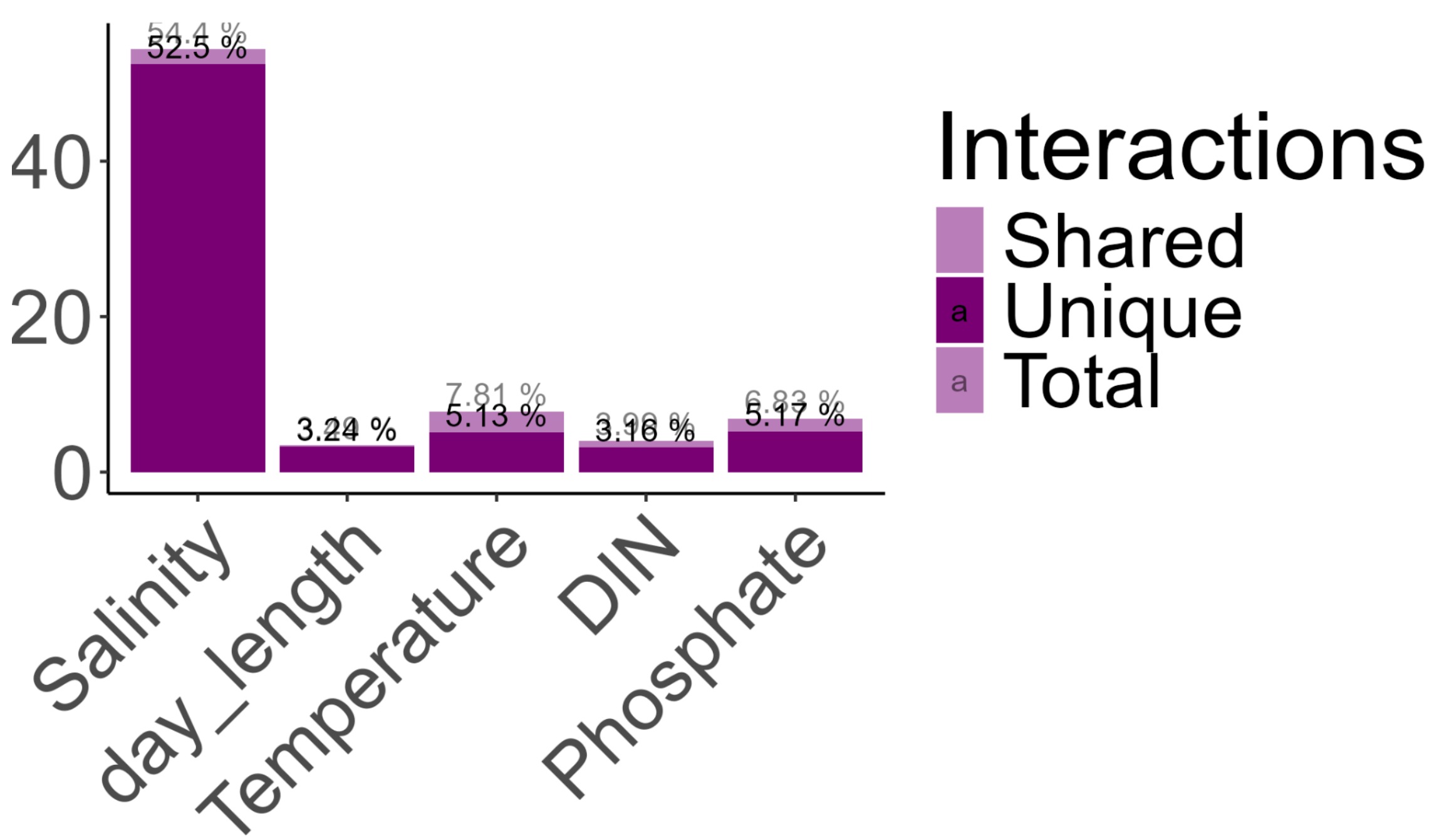

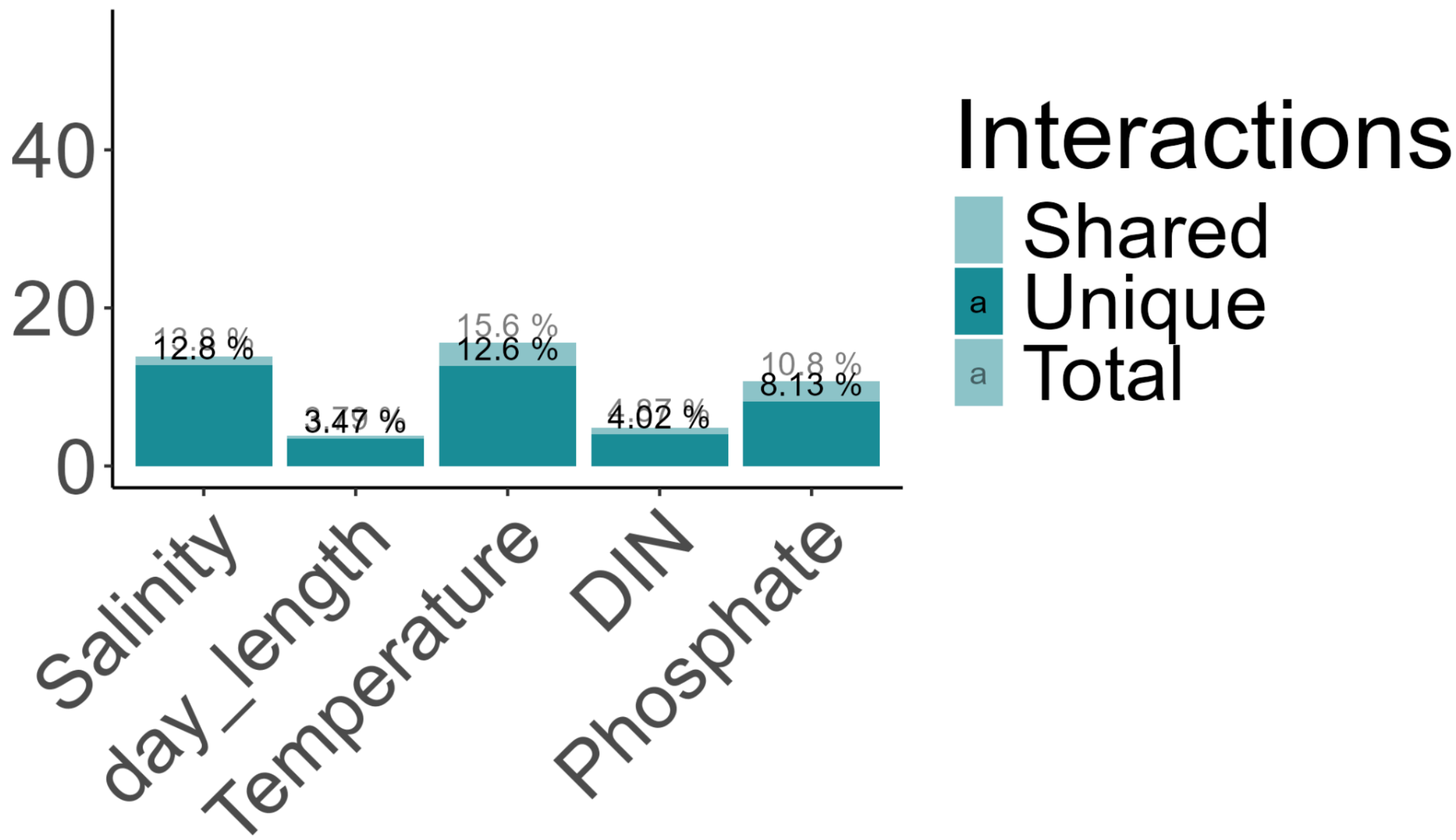

Lines 180–183: If seasonal variation is a primary focus, why was day length selected as the seasonal indicator instead of directly incorporating sampling month or season? This choice should be justified in the Methods section rather than discussed only in the Results.

Reply: We now added the following sentence in the methods section:

"*We chose day length as one of the factors, as it has been connected with seasonal changes in bacterial alpha diversity36,38 and, together with temperature, should catch the full scope of seasonality (as temperature increase is lagging behind day length).*" (lines 644-647)

Lines 197–198: This sentence is unclear and should be rewritten for clarity.

Reply: We rephrased the sentence:

"*We identified microbial families whose abundance most strongly followed these changes in dbOTU-based beta-diversity (Fig. 2c-d).*" (lines 202-203)

Lines 227–228: This statement appears highly speculative and is not directly supported by the presented data. I recommend removing it or providing stronger evidence.

Reply: We added the following sentence with a well-documented example:

"*For example, there is strong evidence for a long-term change in diatom-dinoflagellate ratio during spring bloom in the Baltic Sea (Spilling et al. 2018)*" (lines 233-234)

Lines 229–248: These two paragraphs focus primarily on technical and statistical issues associated with alpha-diversity comparisons between samples collected during 2016–2017 and 2019–2020. There is little biological interpretation.
Unless a stronger ecological explanation can be developed, I suggest substantially shortening or moving this section to the supplementary.

Reply: We have now substantially shortened this section (lines 235-246).

Lines 577–582: This paragraph is confusing because dbOTUs and ASVs appear to be used interchangeably, despite representing different taxonomic units. The terminology should be used consistently throughout the manuscript.

Reply: Thank you for pointing this out. It should have been dbOTUs everywhere. We have now corrected it.

Figure 1b and 1c: Bacterial abundance is typically reported as cells mL^{-1} rather than cells L^{-1}. Consider changing the unit for easier comparison with previous studies. The abundance estimates suggest that bacterial and protistan abundances are of similar magnitude. This is unexpected in most natural aquatic systems, where bacterial abundances are typically orders of magnitude higher than those of protists. Please provide an explanation for this pattern.

Reply: We now explain the pattern in lines 126-132

"*Note that the abundances are given in rRNA gene copies, and as protists often have much higher rRNA gene copy numbers than bacteria(Kembel et al. 2012), they appear similarly abundant despite being less numerous in reality. Indeed, 18S rRNA gene count-derived abundances of phytoplankton classes has been shown to correlate better with biomass than cell counts(Torstensson et al. 2026), and protists have higher biomass per cell and total biomass in the Baltic Sea water column comparable to bacteria (Fridolfsson et al. 2023).*"

We also changed the scale to mL^{-1}. In the process, we found a bug that changed the graph - but not the conclusions - for bacteria in Bothnian Bay and Bothnian Sea, and we also corrected that.

Figure 1d and Figure 3b: These figures are not adequately described in the text. In addition, scaling symbol size by salinity makes the figures unnecessarily busy and difficult to interpret. Since pairwise correlations of environmental variables are already provided in the Supplementary Information, simple boxplots may communicate the results more effectively.

Reply: We have now changed the figures to remove the size-coding of salinity. We do not understand what is missing in the description of the figures. We understand that simple boxplots would convey most of the message, but in our opinion, adding the points provides additional information with little tradeoff for readability. See, e.g., the bimodal distributions of DIN and phosphate in the Bothnian Sea and Skagerrak, which would not be visible in simple boxplots. Although we rarely mention the figure directly, we deem it crucial for critical assessment of the

results and understanding of the context in which they have been obtained, and would prefer to keep it in the main text.

Note: What previously was Fig. 3b is now Supplementary Fig. S7a. This change comes from following the reviewer's comment about decluttering the figures.

Figure 4a and 4b: I do not think it is necessary to scale symbol size according to salinity, given that salinity is already represented on the x-axis.

Reply: We have now removed the size-coding of the symbols in Fig. 4.

Reviewer #2S:

I had a look at the authors' response to reviewer #1 specifically and I feel that the authors addressed this reviewer's concerns appropriately. I also feel that many of the concerns the authors raised around salinity are due to them not accounting for the fact that this study was conducted in brackish water.

Reply: We appreciate the critical assessment of the former reviewer #1's comments and our replies.
