# Supplementary material for: DNA-based monitoring uncovers contrasting salinity-driven diversity patterns of bacterial and protist plankton in the Baltic Sea area
Source: Commun Biol. 2026 Oct 7;9:1308. doi: 10.1038/s42003-026-10977-4 (PMC13646260; doi:10.1038/s42003-026-10977-4)
Supplement: Supplementary file 2 [file 42003_2026_10977_MOESM2_ESM.pdf]

Supplementary figures:

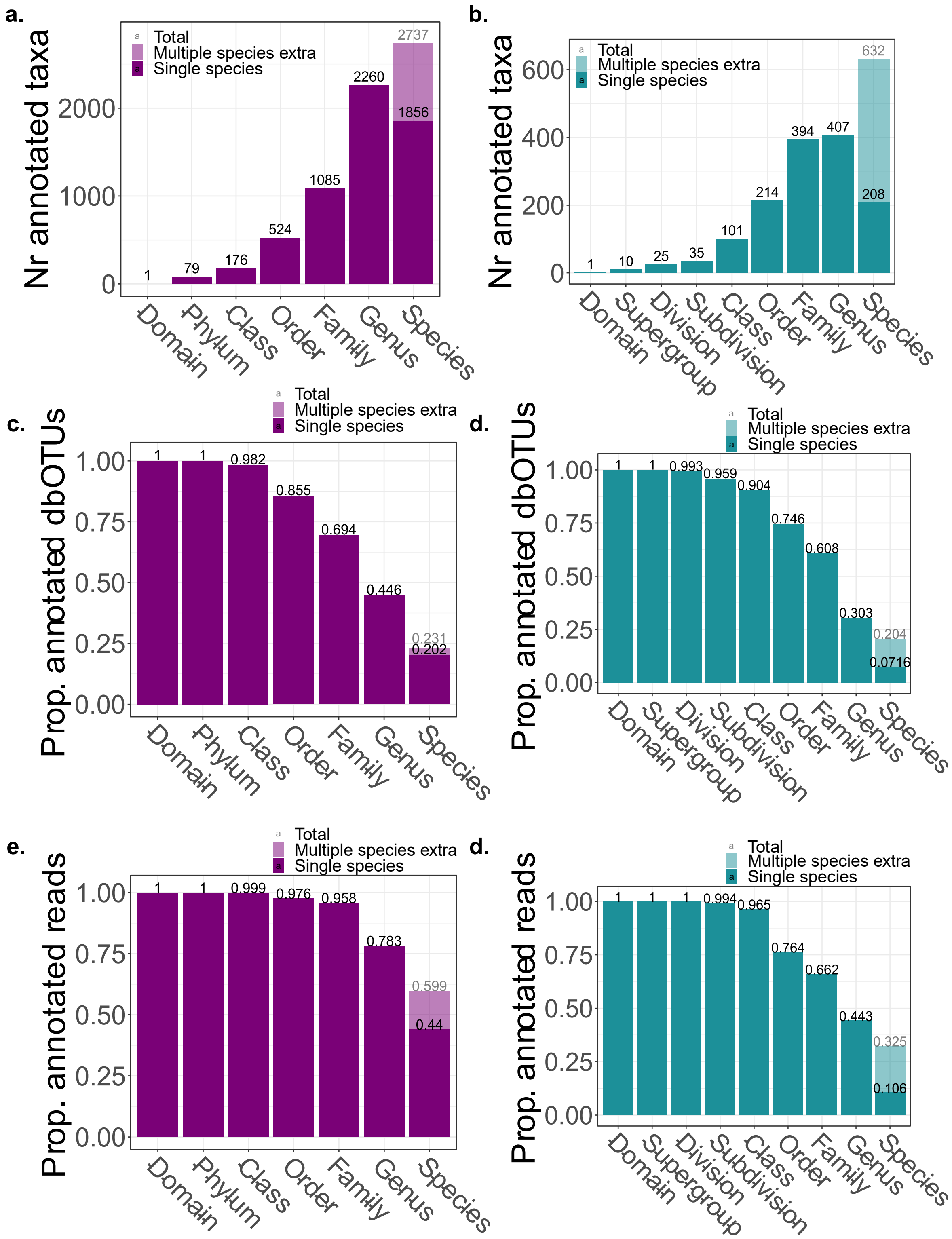

Supplementary Fig. S1 | Taxonomic annotation statistics for the 2019-2020 data a-b. Number of different bacterial (**a.**) and protist (**b.**) taxa found at different taxonomic levels. **c-d**. Proportions of bacterial (**c.**) and protist (**d.**) taxa annotated at different taxonomic levels. **e-f**. Proportions of bacterial (**e.**) and protist (**f.**) reads annotated at different taxonomic levels. "Single species" corresponds to an analysis that provides a species-level annotation only if the query sequence has identical matches to database sequences of a single species. "Multiple species extra" is the extra number/proportion of annotated species when exact matches to multiple species are allowed. Based on 246 samples from the 2019-2020 dataset.

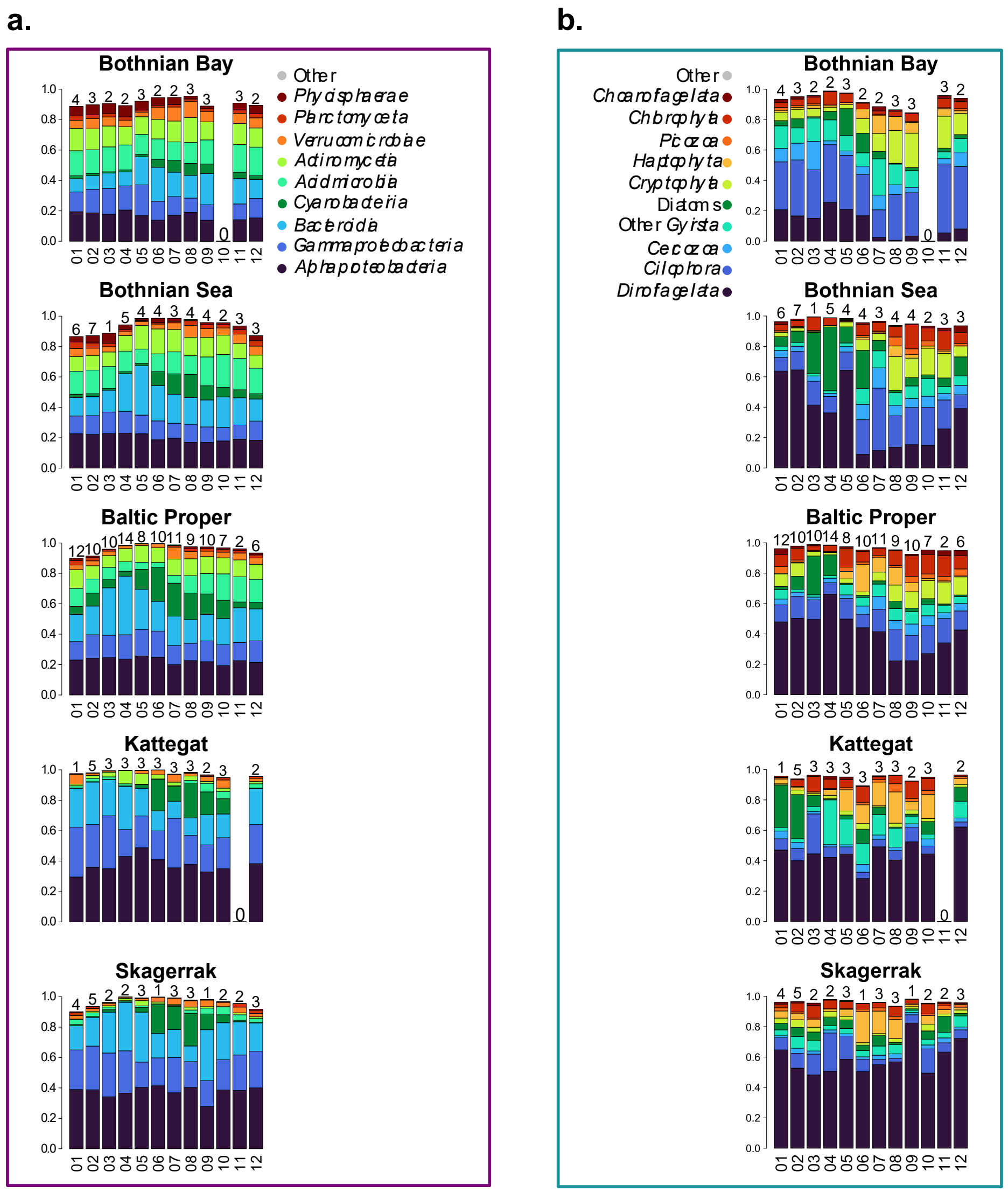

Supplementary Fig. S2 | Spatiotemporal changes in relative abundance of major microbial taxa, 2019-2020 a-b. The proportion of rDNA marker gene reads annotated to **a.** bacterial classes and **b.** protist subdivisions averaged over each month and the Baltic Sea area basin. Only the classes/subdivisions that, on average, corresponded to >0.01 of reads across samples are shown. *Gyrista* has been divided into diatoms and other *Gyrista*. Above each bar, the number of samples collected in the respective month and basin is given.

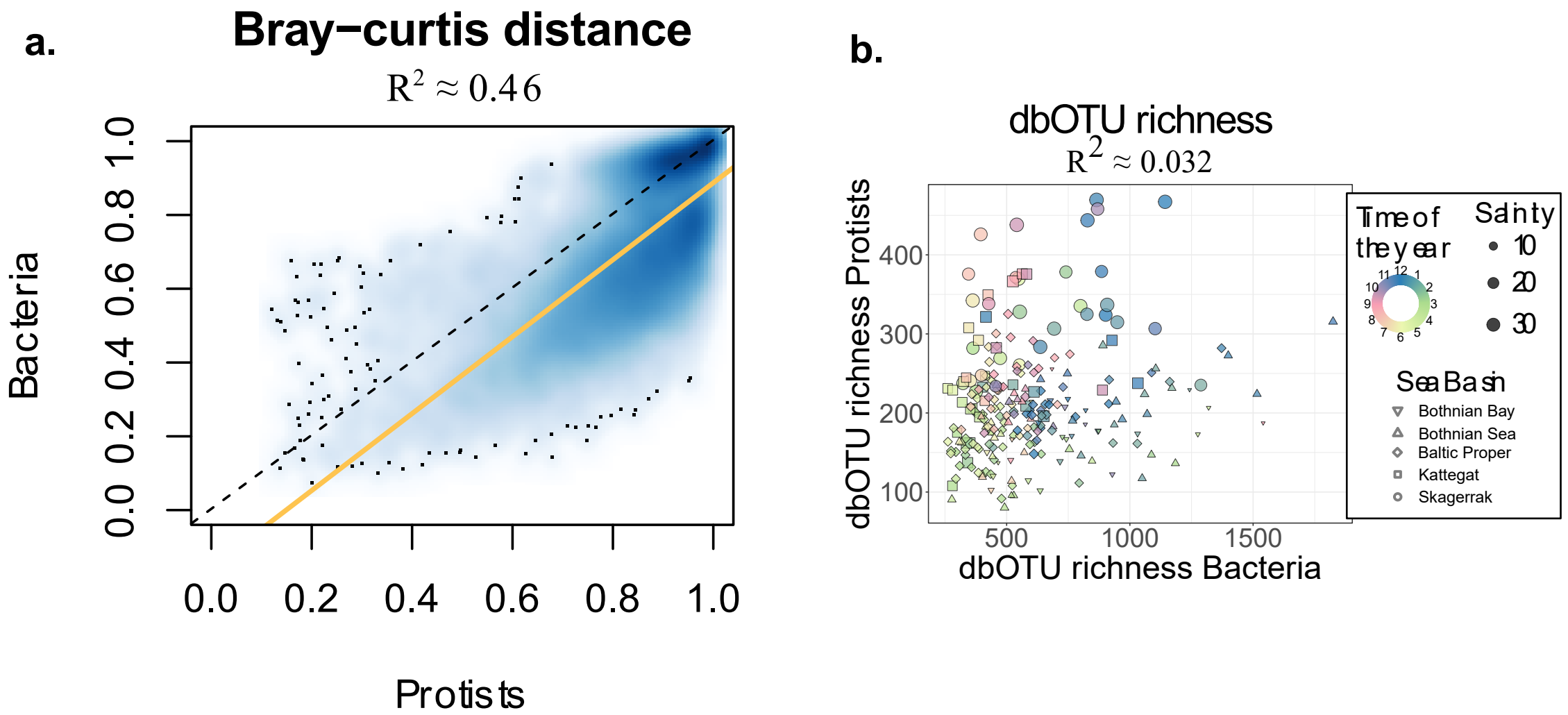

Supplementary Fig. S3 | Dissimilarity of diversity metrics between bacteria and protist, 2019-2020 a. A density map of beta diversity calculated as Bray-Curtis distances. The golden line represents the best linear fit, while the dashed black line is the identity line. **b.** Number of detected dbOTUs (alpha diversity). N=246

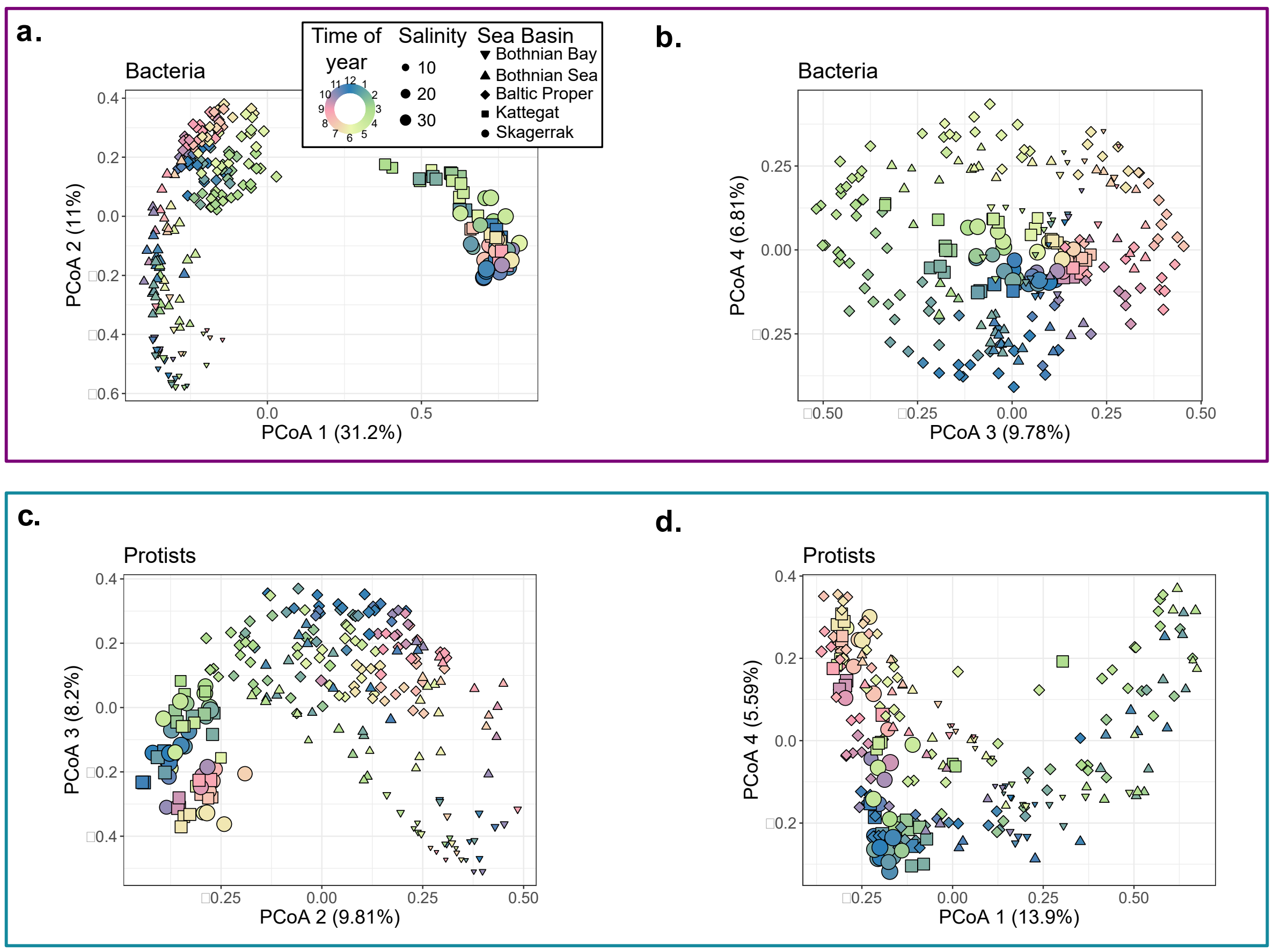

Supplementary Fig. S4 | Principal coordinate analysis (PCoA) 2019-2020. The first four ordination axes are based on Bray-Curtis distances between bacterial (**a-b**) and protist (**c-d**) communities. Note: The axes are grouped and ordered by which ones, after visual examination, correspond to geographic structuring (**a**, **c)** and which ones to seasonal effects (**b**, **d**). This ordering corresponded to showing ordination axes with decreasing variation explained for bacteria (**a-b**). However, for protists, the first plot (**c**) shows the second and the third components, while the second plot (**d**) shows the first and the fourth components. N=246

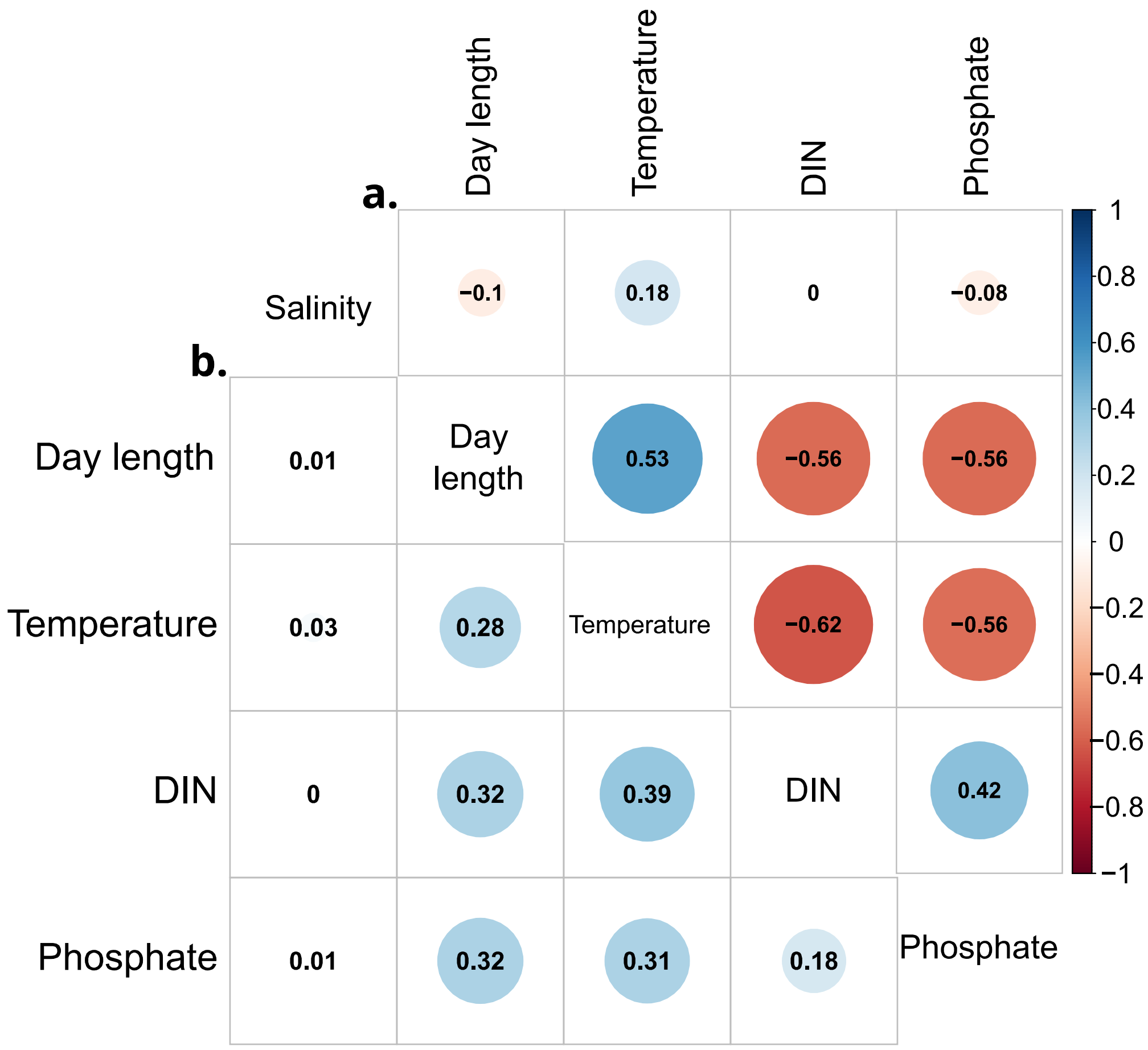

Supplementary Fig. S5 | Correlations between chosen environmental variables for the 2019-2020 dataset **a.** Pearson correlation coefficients (ρ) on an upper-triangle correlation plot. **b.** Coefficients of determination (R^2) on a lower-triangle correlation plot. N=228

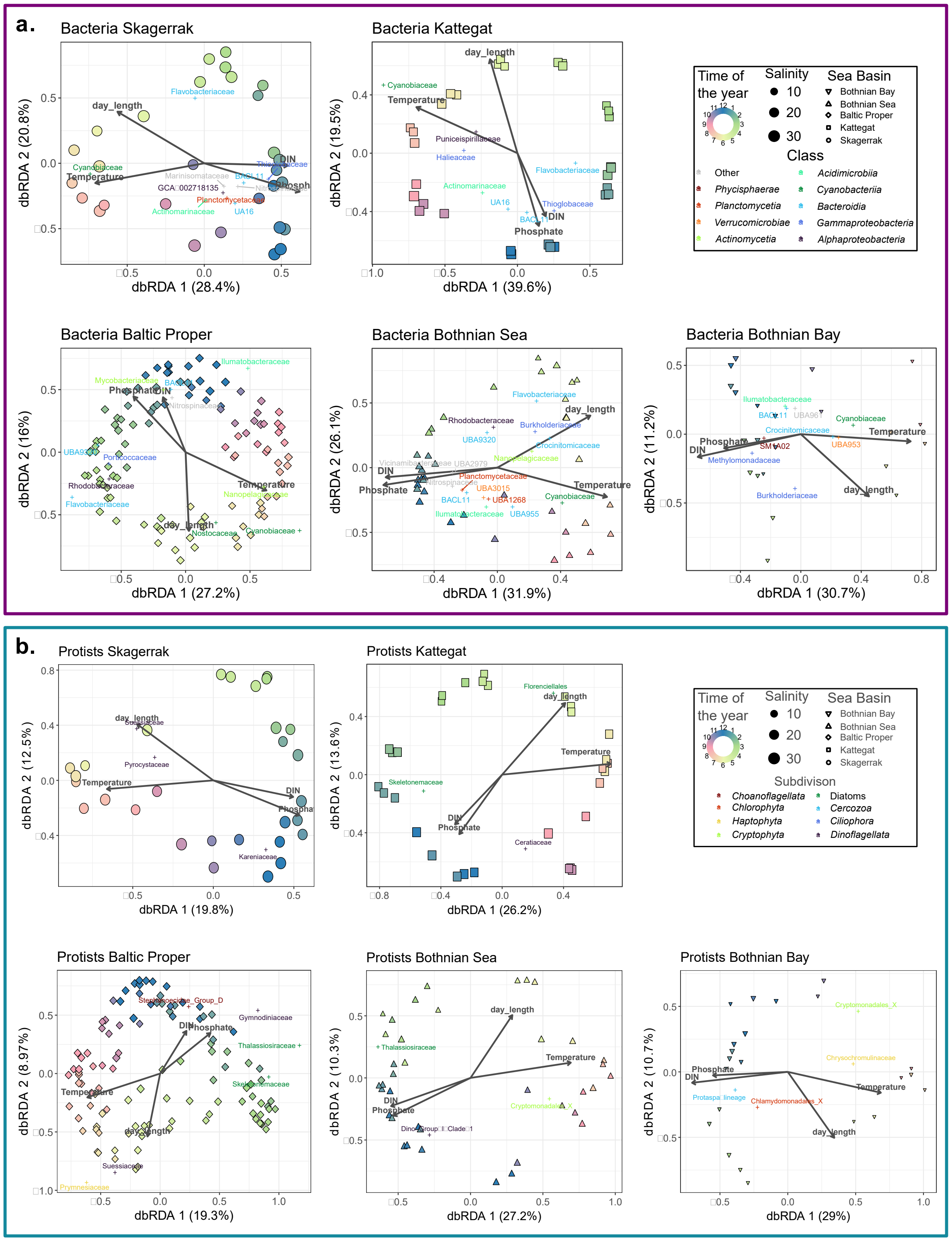

Supplementary Fig. S6 | Seasonal changes in community composition within distinguished basins of the Baltic Sea area. distance-based Redundancy Analysis (dbRDA) based on Bray-Curtis distances between the bacterial (**a.**) and protist (**b.**) communities within each of the distinguished basins of the Baltic Sea area. Weighted averages of families deviating (Bonferroni corrected P < 0.05) were placed on the plot, with values rescaled by a factor of 0.5. The families were color-coded, assigning them to classes/subdivisions which, on average, corresponded to >0.01 of reads across samples are shown (or to the group "Other"). *Gyrista* has been divided into diatoms and other *Gyrista*. Percentages of variation explained by each dbRDA component are given in brackets by the axes labels.

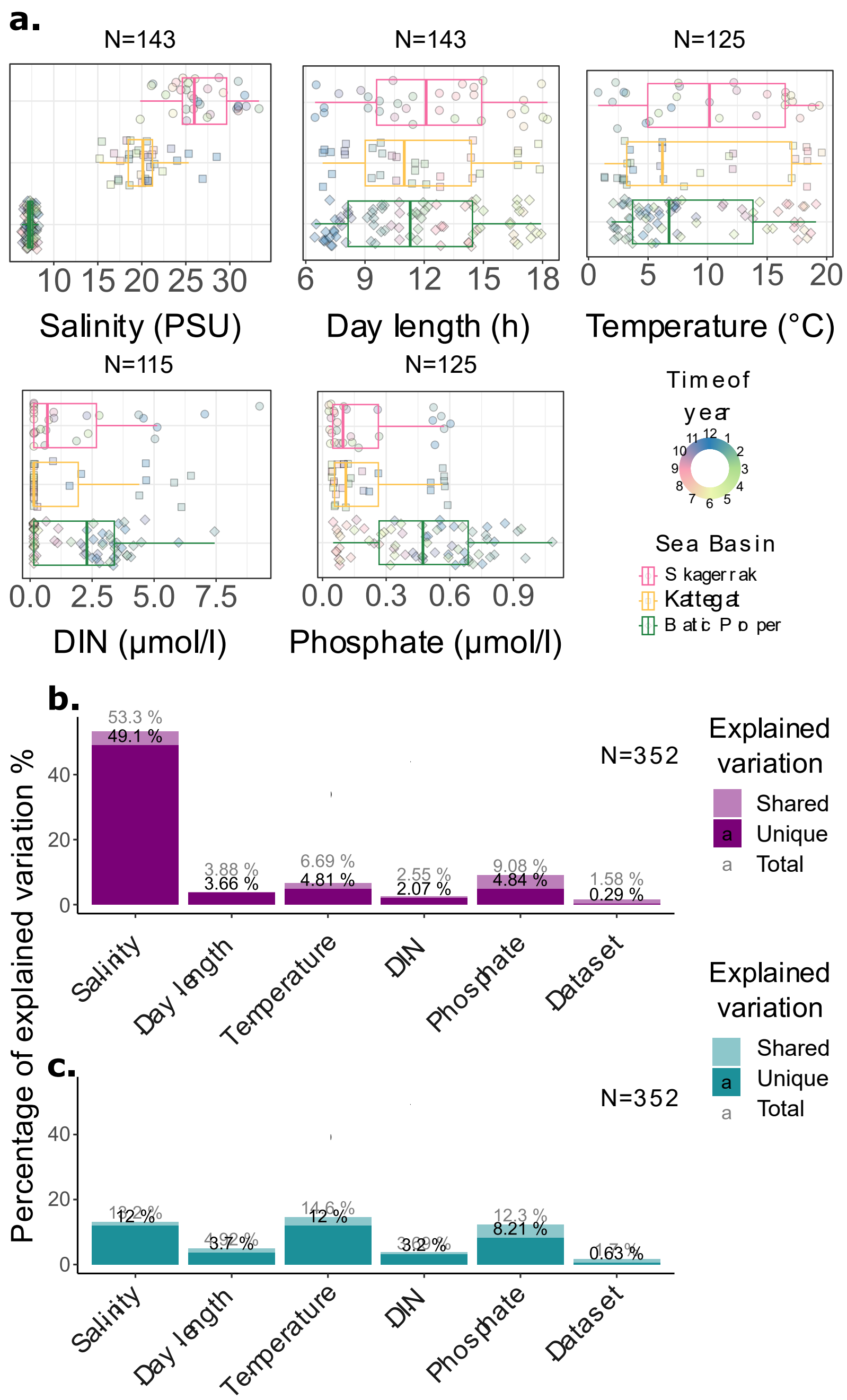

Supplementary Fig. S7 | Distribution of environmental variables for 2015-2017 dataset and variance partitioning across the datasets. a. The distribution of measurements of major physicochemical and geographic parameters for each basin in the 2015-2017 dataset. The lines on the boxplot represent (from the middle) median, 75th percentile, and the whiskers extend to 1.5 times the interquartile range **b-c.** Variance partitioning of bacterial (**b.**) and protist (**c.**) diversity, based on Bray-Curtis distances and dbRDA. Dataset stands for a dummy variable differentiating the samples collected in 2015-2017 and 2019-2020.

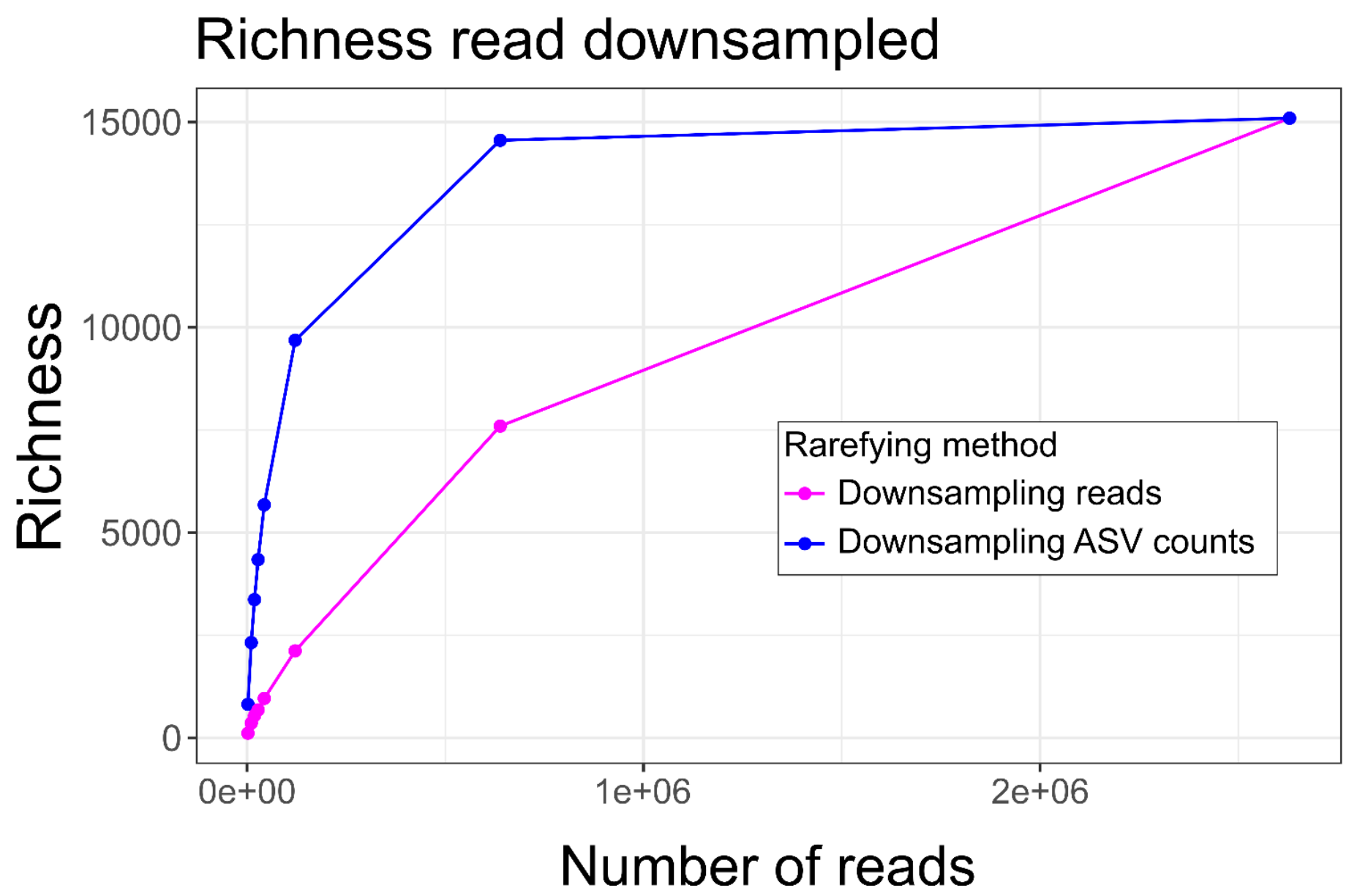

Supplementary Fig. S8 | Downsampling raw reads vs count table-based rarefaction for richness estimation. The ASV richness values obtained based on downsampling the deepest sequenced sample from the 2015-2017 dataset, either at raw read (‘reads’) or ASV count table (‘ASV counts’) level. dbOTUs were not obtained here, as distribution across one sample provides no useful information. The figure is based on 16S data without filtering out plastids and mitochondria.

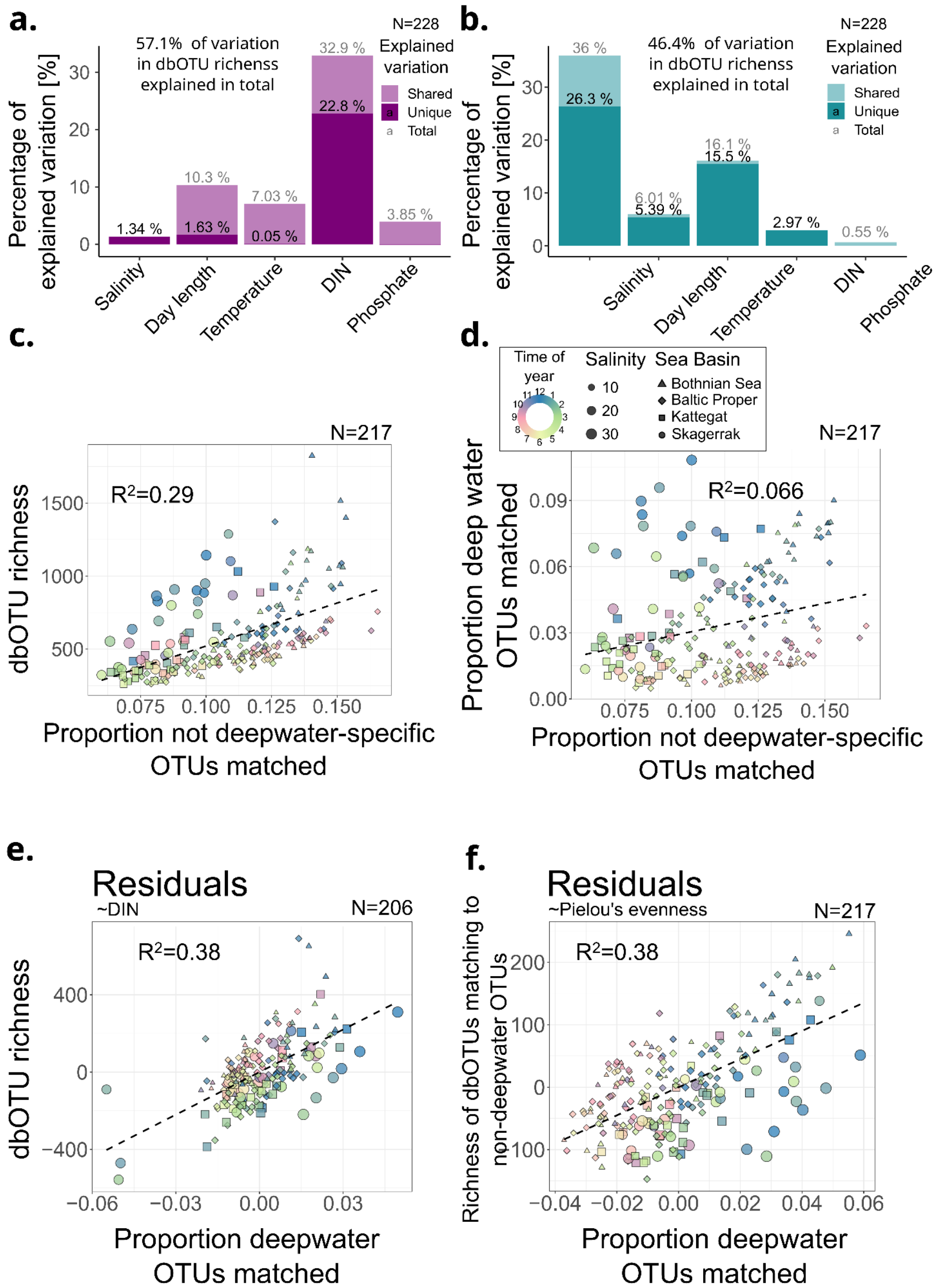

Supplementary Fig. S9 | Bacterial alpha diversity a-b. Variance partitioning of bacterial (**a.**) and protist (**b.**) dbOTU richness. **c.** Proportion of all non-deepwater-specific OTUs ("surface" and "both" categories) having a match to a dbOTU from a sample vs. bacterial dbOTU richness. **d.** Proportion of all non-deepwater-specific OTUs vs. proportion of all deepwater OTUs. **e.** The residuals of proportion of all deepwater OTUs vs. dbOTU richness from models correlating the factors to DIN, corresponding to partitioning out the unvisualized parameter. **f.**The residuals of proportion of all deepwater OTUs vs. dbOTU richness from models correlating the factors to Pielou's evenness. Bothnian Bay was excluded the analysis, since there were no deepwater samples taken in this region in the transect-based study[17]. The same number of samples from the transect-based study[17] (N=106) was used for all the panels (samples not shown, see Methods for details). The same legend applies to all the panels. DIN - dissolved inorganic nitrogen.

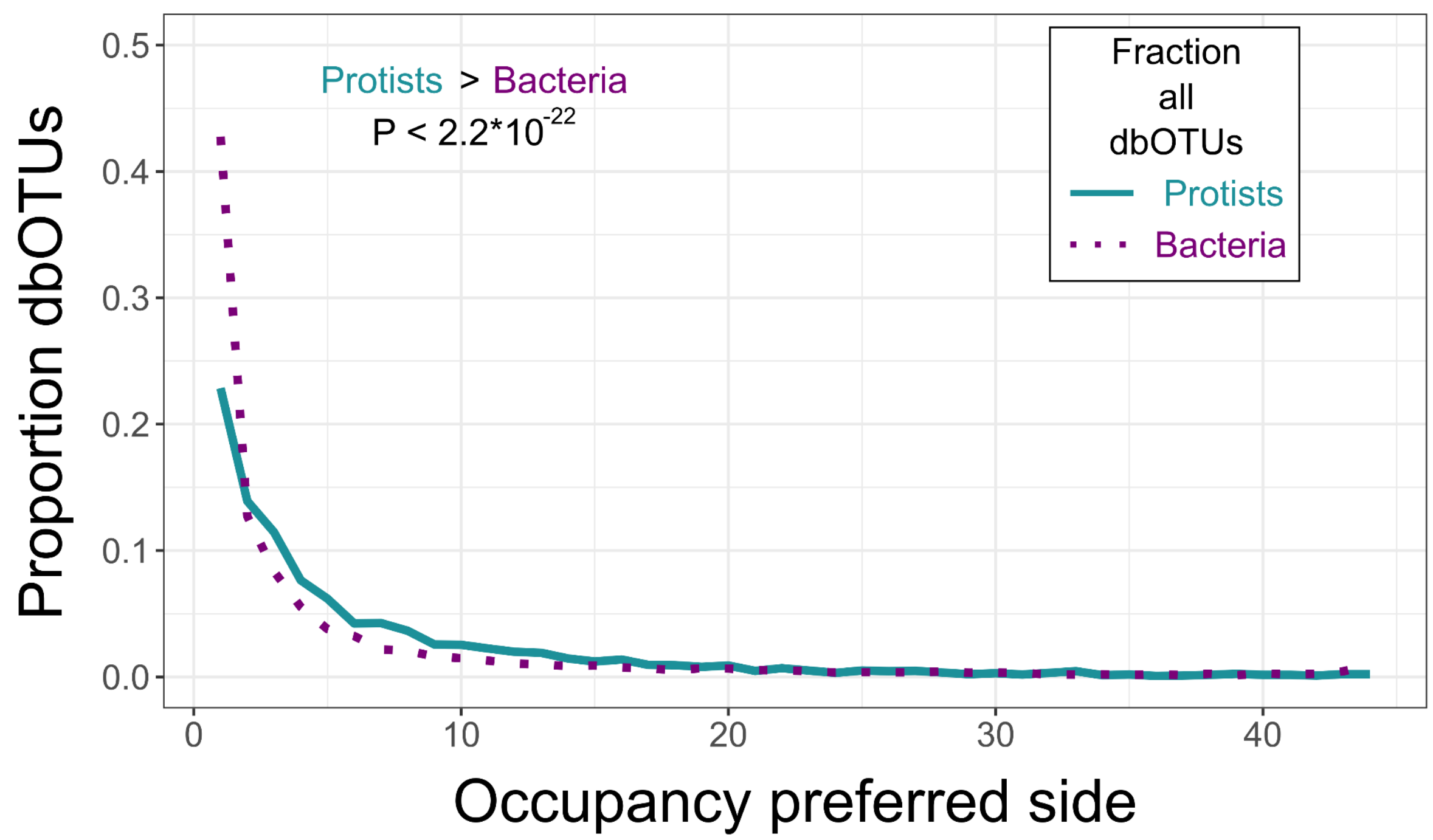

Supplementary Fig. S10 | Distribution of occupancy values on the preferred side of the salinity barrier. The *P*-value comes from Wilcoxon rank-sum test comparing occupancy values between bacteria and protists.

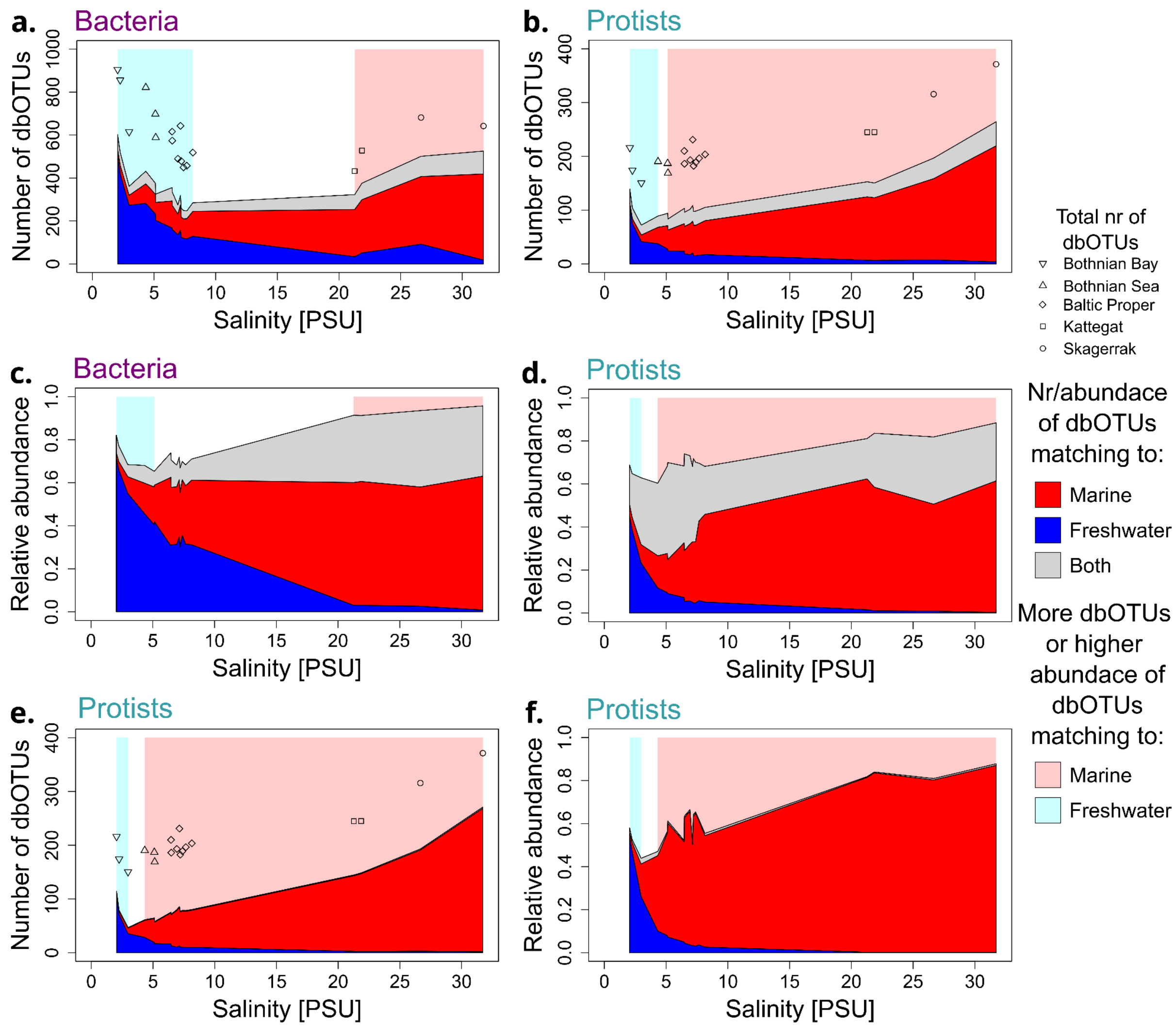

Supplementary Fig. S11 | dbOTUs matching marine and/or freshwater database sequences after equalizing the number of database sequences per biome (a-d.) or using full-length 18S rRNA gene sequences (e-f). a-b. The mean number of bacterial (**a.**) and protists (**b.**) dbOTUs matching marine or freshwater database sequences, or both, after downsampling freshwater (**a.**) or marine (**b.**) database sequences to match the number available for the other biome. Mean total numbers of dbOTUs per station, based on the same rarefied count table, are labeled with sea-basin-coded shapes. The background color indicates salinity ranges at which all the stations had more dbOTUs matching to freshwater or marine database sequences. **c-d.** Relative abundance of bacterial (**c.**) and protists (**d.**) dbOTUs matching marine or freshwater database sequences, or both, after downsampling freshwater (**a.**) or marine (**b.**) database sequences to match the number available for the other biome. **e-f.** The number (**e.**) or relative abundance (**f.**) of protist dbOTUs matching marine or freshwater database sequences, or both, based on all full-length sequences. The common legend for all panels is on the right-hand side.
